# Ultrasound-responsive diagnostic and therapeutic micro-/nanoplatforms for biomedical applications and clinical translation^☆^

**DOI:** 10.1016/j.ultsonch.2025.107524

**Published:** 2025-08-21

**Authors:** Wei Guo, Siying Gao, Yiran Hao, Zijing Li, Haoyuan Hu, Huijun Wu, Changhao Hu, Xueqin Cheng, Weiwen Zhao, Yuxuan Kong, Hong Jiang, Songyun Wang

**Affiliations:** Cardiovascular Hospital, Renmin Hospital of Wuhan University; Cardiac Autonomic Nervous System Research Center of Wuhan University; Cardiovascular Research Institute, Wuhan University; Hubei Key Laboratory of Cardiology, Wuhan 430061, China

**Keywords:** Ultrasound-responsive, Micro-/nanoplatform, Biomedical application, Theranostics, Imaging, Clinical translation

## Abstract

Ultrasound is a widely utilized diagnostic and therapeutic tool in biomedicine due to its non-invasiveness, biocompatibility, cost-effectiveness, and high tissue penetration depth. With the continuous development of materials science and nanomedicine, ultrasound-based diagnosis and treatment have achieved significant advancements. By combining ultrasound as a trigger with responsive biomaterials, platforms with diagnostic and therapeutic performance for specific diseases could be established, offering a promising approach for the development of precise medicine. Through various synthesis and material modification techniques, diverse biomaterials are endowed with diagnostic or therapeutic properties. While ensuring their biocompatibility and biosafety, their tissue targeting specificity is enhanced, thereby minimizing adverse drug reactions. In this review, we introduce the biological effects of ultrasound and the classification of ultrasound-responsive micro-/nanoplatforms, and systematically describe the relevant applications in the diagnostic imaging and treatment of tumors, cardiovascular diseases, infectious diseases, neurological diseases, and metabolic diseases. Finally, we summarized relevant clinical trials in this field and proposed challenges and solutions in the process of clinical translation to better promote the widespread clinical application of this technology and provide novel avenues for the development of clinical diagnostic and therapeutic technologies.

## Highlights

• Classifications of ultrasound-responsive biomaterials.
• Biological effects of ultrasound and ultrasound-responsive biomaterials.
• Biomedical applications of ultrasound-responsive micro-/nanoplatforms.
• Clinical translation and future improvements of ultrasound-responsive micro-/nanoplatforms.

## 1 Introduction

Recently, with the continuous development of precision medicine and personalized healthcare, clinical practice has seen an increasing demand for therapeutic and diagnostic platforms for real-time monitoring of patients' disease progression and the timely delivery of personalized therapeutic measures [1,2]. A range of non-invasive, external physical factor-based platforms have been applied for monitoring the anatomical morphology and physiological functions of target tissues and personalized treatment, in particular light, electricity, magnetism, X-rays, and ultrasound (US) [[3], [4], [5], [6]]. However, optical diagnostic and therapeutic methods have the disadvantage of insufficient tissue penetration and potential damage to normal tissue; electrical stimulation also has the potential to damage non-target tissues and is prone to patient discomfort; magnetic effect applications require an external magnetic field to accurately detect the condition of the lesion site or focus stimulation on the target tissue for diagnosis or treatment, which results in higher costs, larger equipment and complexity of use; and X-rays have the well-known hazards of ionizing radiation [3,6]. In contrast, US has attracted considerable attention due to its excellent depth of tissue penetration, precise focusing capability, no ionizing radiation, non-invasiveness, portability, low cost, and satisfactory biocompatibility. Consequently, it is of considerable clinical translational potential to utilize US to construct a platform for personalized diagnosis and treatment.

With the prosperous development of nanomedicine, the performance of ultrasonic diagnosis and treatment has been continuously improved, injecting new vitality into the development of US-based biomedicine [5]. The application of various US-responsive biomaterials expands the practical scope of US applications. US contrast-enhanced imaging can be achieved through some acoustic contrast agents, enabling real-time monitoring of disease progression utilizing higher spatial and temporal resolution imaging. In addition, combining external ultrasound stimulation and US-responsive biomaterials allows for localized energy conversion or amplification of acoustic energy, resulting in non-invasive and precise modulation of target tissues [4,7]. Recently, there are numerous emerging US-responsive micro-/nanoplatforms equipped with both diagnostic imaging and therapeutic capabilities, achieving dynamic monitoring of disease progression, on-demand drug delivery, and imaging-guided personalized therapy [3,8].

**Fig. 1:**
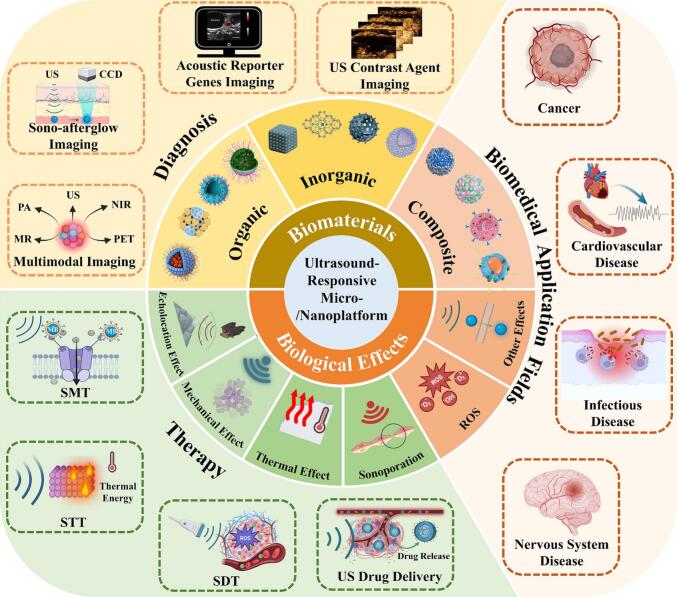
The classification, biological effects, and biomedical applications of ultrasound-responsive biomaterial platforms. US, ultrasound; PA, photoacoustic; CCD, charge coupled device; MR, magnetic resonance; NIR, near-infrared; PET, positron emission tomography; SMT, sono-mechanical therapy; MB, microbubbles; STT, sono-thermal therapy; SDT, sonodynamic therapy; ROS, reactive oxygen species. Reproduced with permission from ref. [84], Copyright 2024, Elsevier. Ref. [43], Copyright 2023, American Chemical Society. Ref. [152], Copyright 2019, Open Access. Ref. [91], Copyright 2019, American Chemical Society. Ref. [87], Copyright 2022, American Chemical Society. Ref. [106], Copyright 2022, Wiley. Ref. [103], Copyright 2022, American Chemical Society. Ref. [104], Copyright 2024, Wiley. Ref. [140], Copyright 2024, Open Access. Ref. [78], Copyright 2022, Wiley. Ref. [124], Copyright 2024, Elsevier. Ref. [123], Copyright 2021, Open Access. Ref. [170], Copyright 2022, IVYSPRING. Created with

Currently, existing reviews mainly focus on the physical features, chemical properties, and advanced synthesis processes of US-responsive micro-/nanoplatforms [3,6]. There are few reviews focusing on the interaction mechanisms between ultrasound and US-responsive biomaterials for diagnosis and treatment, as well as the construction of integrated diagnosis and treatment micro-/nanoplatforms. However, in clinical practice, it is often desired to monitor disease progression in real-time while conducting personalized treatment in the optimal therapeutic window. This necessitates the urgent construction of an integrated diagnostic and therapeutic platform. Therefore, this review aims to provide a systematic and comprehensive overview of the specific applications of US-responsive micro-/nanoplatforms in the biomedical field, specifically in the fields of diagnostic imaging, therapeutic and diagnostic-therapeutic integrated platform construction (Fig. 1). Furthermore, the biocompatibility of US-responsive biomaterials will be discussed in depth, as well as the challenges and improvement measures for their clinical applications, providing novel insights to promote their further clinical transformation.

BioRender.com.

## 2 Biological effects of ultrasound

Ultrasound can modify the structure and properties of materials through a variety of mechanisms such as cavitation, hydrodynamic effects, radiation forces, and thermal effects. These mechanisms provide the basis for the development of smart biomaterials with tunable mechanical properties and transport properties. Examples of innovations include hydrogels and composites, drug delivery systems, and tissue engineering matrices [9]. Meanwhile, ultrasound technology is increasingly used in the biomedical field, and its unique mechanical and thermal effects provide new avenues for disease treatments **(**Fig. 1**)**.

In addition to the direct biological effects of ultrasound itself, the combination of ultrasound and biomaterials can further expand its therapeutic potential. For example, the application of ultrasound combined with sonosensitizers has achieved significant therapeutic effects by inducing sonodynamic effects, and this therapy is becoming a promising treatment in the field of medicine [10]. Ultrasound-responsive drug delivery systems (DDS) have emerged as a potential strategy to overcome the limitations of traditional chemotherapy, demonstrating broad application prospects [11].

### 2.1 Mechanical effects

The mechanical effects of ultrasound primarily stem from its physical vibrational characteristics, achieving biological effects through cavitation and acoustic radiation force. Cavitation refers to the formation of microscopic bubbles (cavitation bubbles) in a liquid by ultrasound, whose periodic expansion and collapse generate localized shock waves and microjets, causing mechanical damage to cell membranes or changes in permeability. For example, cavitation effects have important applications in targeted drug delivery and tissue cutting [12,13]. Meanwhile, the directed pressure exerted by ultrasound on a medium can induce cellular structural deformation or displacement, commonly used in non-invasive neural modulation or focused ultrasound ablation [13,14]. For instance, acoustic radiation force activates mechanosensitive ion channels (e.g., Piezo1/2) by inducing mechanical deformation of cell membranes, triggering calcium ion influx and regulating neuronal activity [12,14]. Low-frequency ultrasound (<1 MHz) and moderate intensity (0.4–1.5 W/cm^2^) are more likely to elicit mechanical effects [15,16], while short pulse stimulation (<1 s) and low duty cycles (<40 %) can reduce heat accumulation and enhance the dominance of mechanical effects [13,14]. Another study showed that the mechanical effects of ultrasound could not only directly alter cell membrane structure to promote substance transport but also indirectly achieve therapeutic effects by influencing the cytoskeleton [17], regulating vascular permeability, and modulating endocytosis. These mechanical effects collectively provide the theoretical foundation and technical means for developing new therapeutic methods.

Ultrasound can produce mechanical effects by stabilizing the formation and rupture of bubbles, which in turn destroy carriers such as specific micro-/nanoplatforms and release encapsulated drugs. By using Biocompatibility materials as carriers, therapeutic drugs can be released or activated by specific protonation, hydrolysis, phase transitions, and molecular or supramolecular conformational changes. In this process, pressure oscillations generated by ultrasound affect the homeostasis of the drug carrier and are accompanied by mechanical and thermal effects that further alter its conformation [11].

### 2.2 Thermal effects

Thermal effects involve the conversion of ultrasound energy into thermal energy, with high-intensity focused ultrasound (HIFU) being a typical example. Its localized high temperatures (>65 °C) can induce protein denaturation, making it suitable for tumor ablation [12,16]. The propagation of ultrasound waves in tissues leads to viscous absorption and molecular relaxation processes [15,16], converting energy into thermal energy. Temperature-sensitive ion channels play a key role in the thermal effect [12,14]. For instance, TRPV1 mediates the release of inflammatory factors or neural signaling by sensing local temperature increases (>43 °C). Additionally, the thermal effect of ultrasound holds potential in immunotherapy and tumor immunogenic cell death (ICD) [18].

The thermal effects generated by the propagation of ultrasound in tissue can also activate temperature-sensitive micro/nanoplatforms. Ultrasound-responsive thermosensitive drug delivery systems can utilize this characteristic to achieve therapeutic effects. At physiological temperatures, the system remains stable. When exposed to ultrasound, the local temperature rises, causing phase changes or lipid conformation changes, thereby releasing the loaded drugs [11].

### 2.3 Sonodynamic effects

Sonodynamic therapy (SDT) is an emerging treatment strategy combining US and sonosensitizers. In recent years, SDT has garnered significant attention for the treatment of cancer, infectious diseases, and cardiovascular disorders due to its deep tissue penetration, absence of ionizing radiation, excellent controllability, enhanced therapeutic efficacy, and favorable biosafety profile [3,19,20]. The therapeutic effect of SDT mainly depends on reactive oxygen species (ROS), which is capable of damaging key cellular components such as proteins, lipids, and nucleic acids, ultimately leading to autophagy, programmed cell death or necrosis. The mechanisms of SDT can be understood from two main aspects. The first potential mechanism is sonoexcitation. In the presence of ultrasound, sonosensitizers absorb energy and undergo an electron transition from the ground state to the excited state. When returning to the ground state, the released energy reacts with surrounding oxygen molecules to generate ROS. Secondly, the cavitation effect of ultrasound causes rapid pressure changes in the medium, forming microbubbles. These bubbles undergo cycles of growth and collapse under ultrasound, generating sono-luminescence and thermal decomposition during rupture, which further promote ROS production and ultimately induce damage to target cells [3,21].

In the complex pathological environment, there is a significant synergy among mechanical effect, thermal effect and sonodynamic effect. Firstly, mechanical effects, such as microbubble rupture, may enhance drug delivery and cell membrane permeability, promoting the enrichment of sonosensitizers in tumor tissues, thereby improving the SDT effect [10,18]. At the same time, thermal effect can improve the hypoxic state of tumor by inducing vasodilation and increasing blood flow [16]. It can enhance the efficiency of ROS production by improving the hypoxic state of the tumor microenvironment, thereby overcoming the limiting effect of hypoxia on SDT [10]. For example, nanosystems that combine thermal effects with oxygen release (such as MPDA-GOx@PFP@O_2_) can effectively enhance the therapeutic response of hypoxic tumors [22]. In addition, mechanical and thermal effects can also produce synergistic effects to promote the therapeutic efficacy of SDT. For example, a segmented ultrasound strategy can be used, in which low-intensity ultrasound is first used to open the vascular barrier through mechanical effect, and then thermal effect is used to stabilize blood flow, thereby synergistically improving vascular permeability and the efficiency of drug targeted delivery [13,17]. The combination of the three provides an effective way to break through the limitations of SDT in drug delivery and oxygen dependence.

### 2.4 Sonoporation

Sonoporation refers to the utilization of ultrasound waves to induce the temporary formation of reversible pores in the cell membrane, thereby allowing external substances such as drugs or genes to enter the cell [23]. This process not only improves the efficiency of the therapeutic substance to enter the target cell, but also reduces the side effects on normal tissues. In terms of sonoporation, microbubbles and nanobubbles are often used as carriers. Under ultrasonic irradiation, they can release drugs or genetic material through their mechanical effects or the production of ROS, thereby promoting the uptake of substances by cells to achieve therapeutic effects [23,24].

### 2.5 Other biological effects

Ultrasound can also be integrated with other physical phenomena, such as sono-optical, sono-mechanical effects, sono-piezoelectric effects and sono-thermal effects, enabling multimodal therapeutic approaches. For instance, ultrasound can stimulate luminescence in certain biomaterials [25,26], allowing deep tissue penetration and precise energy delivery to target regions—offering powerful tools for bio-imaging, drug delivery, and tumor therapy. The synergistic application of ultrasound and biomaterials can amplify the mechanical or thermal effects of ultrasound in specific anatomical regions, and can also be converted into optical or electrical stimuli through various physicochemical reactions, thereby achieving efficient disease treatment. This method shows particular promise in fields such as oncology, cardiovascular disease, neuromodulation, and bone regeneration [[27], [28], [29], [30]]. Collectively, ultrasound serves as a versatile tool with excellent therapeutic and diagnostic capabilities on its own. However, when integrated with ultrasound-responsive biomaterials and technologies, its medical applications expand dramatically through multiple synergistic biological effects. In order to maximize the synergistic effects of these factors and mitigate potential adverse impacts, it is necessary to formulate personalized treatment regimens tailored to specific pathological conditions and individual variations. For example, optimizing ultrasonic parameters (such as frequency, power, and pulse cycle), and leveraging responsive materials for accurate targeting, etc [14,18].

## 3 Construction of ultrasound-responsive micro-/nanoplatforms

Ultrasound-responsive micro-/nanoplatforms offer the potential for more precise, efficient, and controllable therapies tailored to various disease characteristics and diagnostic/treatment needs. These biomaterials can be surface-modified to enhance targeting or designed with specific phase-transition properties at certain temperatures, pH levels, or pressures to optimize their performance **(**Fig. 1**)**.

### 3.1 Microbubbles (MBs) and nanobubbles (NBs)

As clinical contrast agents, MBs typically consist of a shell made of phospholipids, polymers, or surfactants, encapsulating stable gases like fluorocarbon or sulfur hexafluoride. MBs generally range from 1 to 10 µm in diameter, enhancing ultrasound imaging clarity and facilitating drug or gene delivery through cavitation. However, their relatively large size restricts their penetration into dense tissues, such as across the blood–brain barrier. While NBs typically range from tens to hundreds of nanometers in diameter, offering a higher specific surface area and superior penetration capabilities, making them particularly suitable for drug delivery to deep tissues. Moreover, NBs can be formed *in vivo* from MBs under ultrasound stimulation or synthesized directly. For example, Zhao et al. designed porphyrin microbubbles loaded with FOXA1, combining photodynamic therapy with FOXA1 knockout gene therapy, and applied ultrasound-targeted microbubble destruction (UTMD) technology under contrast-enhanced ultrasound (CEUS) guidance to release photosensitizers and siRNA. Additionally, stimulation with low-frequency pulsed ultrasound can convert this micrometer-scale biomaterial into nanoparticles, significantly enhancing siRNA transfection efficiency and photosensitizer uptake, thereby demonstrating exceptional therapeutic efficacy against breast cancer [31].

### 3.2 Droplets

Droplets are minute particles of a liquid core dispersed under ultrasound shear, stabilized by polymers, surfactants, etc. They are more stable *in vivo* than bubbles, extending drug circulation time, yet their smaller core volume usually limits their drug loading capacity [32]. Just like natural bubbles, droplets also require surface modification to improve targeting. For instance, Liu et al. engineered chitosan/perfluoro hexane nanodroplets (CNDs) to electrostatically complex DDK-2 pDNA via cationic charge-mediated binding, and the ultrasound-triggered CND rupture releasing therapeutic pDNA can significantly inhibit human prostate cancer cell proliferation [33].

### 3.3 Liposomes

Liposomes are composed of a phospholipid bilayer, structurally similar to cell membranes, and possess excellent biocompatibility and stability. They can encapsulate both hydrophilic and lipophilic drugs. Under ultrasound, the thermal effect increases the fluidity and permeability of the liposomal membrane, while the mechanical effect leads to cavitation and drug release [34,35]. Utilizing these features, Kee et al. effectively attenuated neointimal growth by utilizing pioglitazone-loaded nitric oxide echo liposomes (ELIPs) conjugated with anti-intercellular adhesion molecule-1 antibodies, integrated into an ultrasound-core intravascular catheter system [36]. While Gao et al. prepared irinotecan loaded MB-oxaliplatin loaded liposome conjugate (IRMB-OxLipo) and found that ultrasound-delivered IRMB-OxLipo resulted in tumors that were 136 % smaller than those treated conventionally [37]. However, due to their similarity to cell membranes, liposomes often require surface modification to improve targeting.

### 3.4 Polymers

Polymeric materials are typically thicker and more rigid than bubbles, droplets, and liposomes. They can be engineered to form anisotropic shells, which helps improve *in vivo* circulation and drug penetration [3]. Although their biocompatibility might be slightly lower than lipid-based materials, polymers offer higher controllability, design flexibility, and diversity in terms of structure and function. Furthermore, polymers can be designed with temperature, pH, or ultrasound-responsive properties. Polymeric materials can be used to construct various structures, including hollow vesicles, micelles, or hydrogels. Exemplifying this approach, Wei et al. used the block copolymer poly(glutamic acid)-block-poly(ε-caprolactone) (PGA-b-PCL) to develop a kind of MnO_2_-modified polymeric vesicles co-loaded with sonosensitizer Ce6 (designated as Ce6-MnO_2_-PVs), which catalyzed H_2_O_2_ decomposition in the tumor microenvironment to generate O_2_, mitigating hypoxia and achieving 94 % tumor volume suppression [38].

### 3.5 Metals or other inorganic materials

Inorganic materials are relatively simple to synthesize and exhibit excellent chemical and physiological stability, along with multifunctionality. They often act as sonosensitizers, producing ROS to induce cellular apoptosis, and achieve drug delivery and cell killing through various mechanisms, including thermal effects, mechanical effects, and cavitation. Common inorganic materials include metal or metal oxide materials (e.g., gold nanoparticles, titanium dioxide (TiO_2_)), silicon materials, and carbon materials (e.g., fullerenes, carbon nanotubes, graphene, carbon quantum dots, etc.). In 2020, Kim et al. engineered polyphenylboronic acid encapsulated TiO_2_ nanoparticles (pPBA@TNP-DOX). Upon ultrasound irradiation, TiO_2_-mediated ROS generation triggered spatially controlled DOX release, significantly potentiated tumor growth suppression in murine xenograft models [39].

Additionally, various biomaterials can be assembled to form composite materials with superior performance and more abundant functions to satisfy more biomedical application needs. In summary, ultrasound-responsive biomaterials can enhance the efficiency and targeting of drug delivery. Future efforts need to focus on optimizing material composition and structural design, improving the biocompatibility and good degradability of biomaterials, and promoting their safe and effective clinical use.

## 4 Biomedical applications

### 4.1 Diagnostic applications

#### 4.1.1 Ultrasound contrast-enhanced imaging

Ultrasound diagnostic imaging has gained widespread clinical application based on unique advantages such as real-time non-invasiveness and excellent penetration depth **(**Table 1**)**. The introduction of ultrasound contrast agents with signals different from those of human tissues can further enhance the imaging contrast and quantify the morphological and functional changes of human organs more accurately [3]. Based on the different components of the contrast agent shell and contents, they can be classified into organic molecular contrast agents (e.g., microbubbles, nanobubbles, and nanodroplets) and various types of inorganic molecular contrast agents. Meanwhile, the emergence of a series of new image acquisition techniques, such as pulse inversion, singular value decomposition, and contrast agent pulse sequencing, can further take advantage of the high spatial and temporal resolution of contrast agents [40].

**Table 1:** Diagnostic applications of ultrasound-associated responsive biomaterials.

Biomaterials	Material Type	Disease Model	Administration Route	Imaging Features	Diagnostic Application	Ref.
Magnetic navigation microbubbles	Microbubbles	Wistar rat	i.v.	Ultrasound signal significantly increased	Cardiac and great vessel imaging	[42]
E-Cad and N-Cad targeted nanobubbles	Nanobubbles	Lung cancer mouse	i.v.	Tumor cell targeting	Monitoring tumor progression and assessing metastasis	[138]
FN3-linked nanobubbles (FN3_hPD-L1_-NB)	Nanobubbles	CT26 colon cancer mouse	i.v.	Tumor targeting; Signal increased by ∼ 3 times	Monitoring tumor progression	[139]
IR783-SiO_2_NPs@NB	Nanobubbles	Nude mice bearing VX2 tumors	i.v.	Tumor targeting; Contrast and stability enhanced	Monitoring tumor progression	[140]
BiF_3_@PDA@PEG (BPP)	Bismuth-based nanoparticles	Mice with breast cancer and mice with melanoma	i.t.	Display tumor microenvironment pH value	Displaying tumor microenvironment and detecting tumor	[44]
Blinking Nanoparticles (BNPs)	Porous silicon Nanoparticles	Nude mouse	i.m.	Ultrafast background-free imaging	Used for imaging various diseases including cancer	[43]
Acoustic reporting gene system	Blister	Ovarian tumor mouse	s.c.	Significantly improved imaging resolution	Visualising microbial cells deep within the mammalian body	[45]
Trianthracene derivative-based nanoparticles (TD NPs)	Organic nanoparticles	Brain tumor and pancreatic Adenocarcinoma mouse	i.v.	Delayed ultrasound activation; Molecular luminescent imaging mode	Reducing background interference and precise imaging	[50]
Radio-afterglow nanoprobes (RANPs)	Organic nanoprobes	Peritoneal tumor mouse	i.v. & s.c.	Penetration depth up to 15 cm	Auxiliary tumor diagnosis and theranostics	[49]
RGD-SPION-MB	Targeted microbubbles	Breast cancer mouse	s.c.	Targeting α_v_β_3_ integrin receptor	Ultrasound/MRI multimodal imaging; Displaying neoangiogenesis in malignant tumor tissue	[54]
GLANCE	Gold nanorods	Breast cancer mouse	i.v.	Enhanced ultrasound imaging	Realizing ultrasound/photoacoustic multimodal imaging with strong contrast enhancement	[55]

Abbreviation: i.v., Intravenous injection; s.c., Subcutaneous injection; i.m., Muscle injection; i.t., Intratumoral injection.

##### 4.1.1.1 Organic molecular contrast agents

**Fig. 2:**
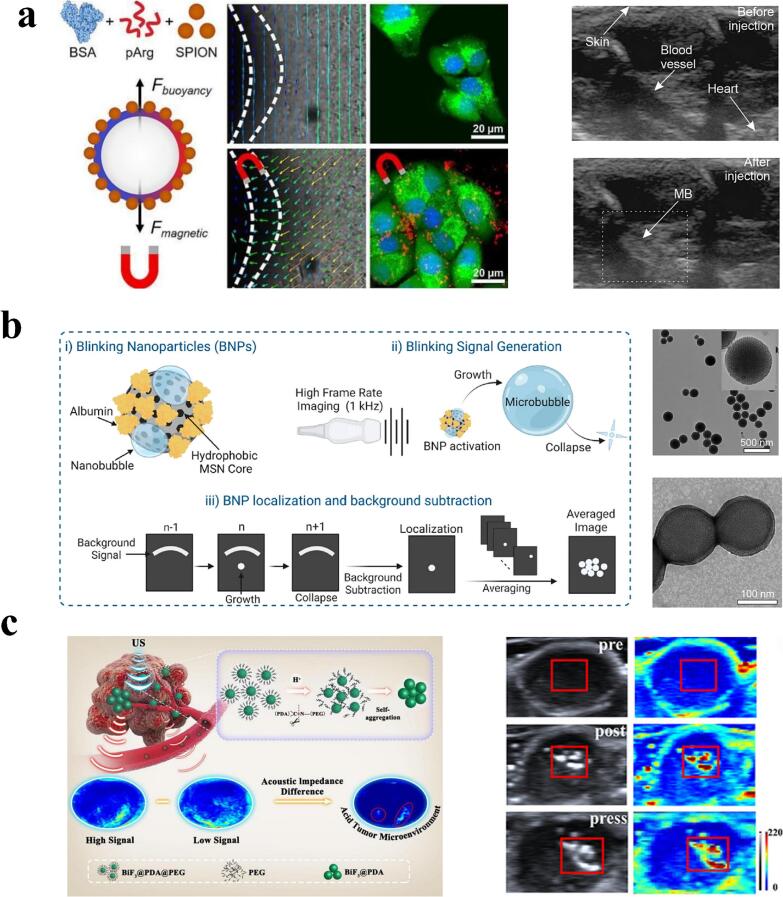
Imaging applications of ultrasound combined with contrast agents. a) Synthesis process of magnetic navigation microbubbles and schematic diagram of magnetic localization imaging in mouse renal cancer cell lines; US images of the heart region before and after MB injection. Reproduced with permission from ref. [42], Copyright 2024, Elsevier. b) Schematic of BNP design, signal localization; porous structure of nanoparticles; diagram of albumin-coated nanoparticles. Reproduced with permission from ref. [43], Copyright 2023, American Chemical Society. c) Schematic representation of BPP as a smart nano UCA for acidic TME sensitive diagnostics; US images of BPP nanoparticles in tumor. Reproduced with permission from ref. [44], Copyright 2022, American Chemical Society.

Currently, the most clinically recognized for effectiveness in organic molecular contrast agents are microbubble contrast agents, now approved by the United States Food and Drug Administration (FDA), to assist clinical ultrasound imaging of the heart and liver [41]. However, shortcomings such as insufficient targeting, short cycle times, and limited structural control hamper further clinical applications of MBs [2]. Based on these challenges, Gusliakova et al. developed a magnetic navigation MB, which was functionalized with superparamagnetic iron oxide nanoparticles to precisely target mouse renal cancer cell lines under magnetic field gradients. At the same time, bovine serum albumin (BSA) and poly-L-arginine (pArg) were added to the MB shell to enhance the stability and acoustic responsiveness of the MB, which dramatically improved the ultrasound imaging signals [42] **(**Fig. 2**a)**.

##### 4.1.1.2 Inorganic molecular contrast agents

Microbubbles, exemplified by SonoVue (Bracco, Milan, Italy), demonstrate promising clinical applications due to their high biocompatibility. However, these organic contrast agents suffer from limited stability and single functionality, with imaging requiring delays of several seconds to minutes, making accurate real-time visualization unattainable. These shortcomings have significantly hindered the widespread adoption of microbubble-based organic contrast agents in various clinical scenarios. In contrast, artificially designed inorganic materials, such as the scintillating Blinking Nanoparticles (BNPs) with particle size less than 200 nm constructed by Sabuncu's team using inorganic mesoporous silica nanoparticles, combine the advantages of strong stability and real-time imaging to make up for the shortcomings of organic contrast agents. The BNPs can generate micron bubbles that rapidly collapse under the activation of low-intensity pulsed ultrasound to achieve microsecond background-free imaging with the help of the acoustic cavitation effect. BNPs can be activated by low-intensity pulsed ultrasound to generate micron bubbles that rapidly collapse, enabling background-free imaging in microseconds with the help of acoustic cavitation [43] **(**Fig. 2**b).** Meanwhile, inorganic molecular materials have the potential for clinical innovation based on their diversity and properties, and ease of editing and modification. Meng's team has proposed an innovative self-enhanced acoustic impedance difference strategy for detecting acidic tumor microenvironments by constructing high-density bismuth-based nanoparticles, BiF_3_@PDA@PEG (BPP). Dispersed individual BPP nanoparticles have little effect on ultrasound imaging. In an acidic tumor microenvironment (TME), however, BPP switches from hydrophilic to hydrophobic, which leads to the aggregation of high-density stone analogs in tumor tissues, forming a large acoustic impedance difference from normal tissues, which visually suggests the pH value of the TME [44] **(**Fig. 2**c).**

##### 4.1.1.3 Acoustic reporter genes for noninvasive imaging

Using genetic engineering, the Bourdeau RW team developed a novel ultrasound contrast agent system for acoustic reporter genes for noninvasive imaging. They created hybrid gene clusters from Bacillus megaterium and Cichlidium aquaticum to enable heterologous expression of air bubbles in *Escherichia Coli (E. coli)* and *Salmonella typhimurium (S. typhimurium),* allowing for non-invasive ultrasound imaging of murine gastrointestinal tracts and tumors with high resolution (<100 μm) and low bulk density (<0.01 %) [45]. This system allows for multiplexed imaging of various microbial cells deep within mammalian tissues using different acoustic reporter genes. More recently, Howells et al. developed a drug-selective acoustic reporter gene system for ultrasound imaging of human cells, significantly improving HEK293T cell imaging signal-to-noise ratio and heterologous expression efficiency, with broad potential applications [46].

#### 4.1.2 Ultrasound-induced luminescence

Ultrasound-induced luminescence is another innovative technology in the fusion of optical and acoustic imaging, which is a multimodal imaging technology that uses pulsed ultrasonic mechanical stimulation to induce afterglow probes to produce long-lived (microsecond to second) afterglow luminescence for optical imaging [47]. Focused ultrasound induces the probe to continue releasing photon signals even after the termination of ultrasound irradiation through the acoustic cavitation effect or piezoelectric effect, and avoids background interference during ultrasound excitation with the help of time-delayed detection technology, which breaks through the limitations of traditional optical imaging [48,49]. Compared with near-infrared PAI, this technology has clinical translational advantages such as high signal-to-noise ratio and sensitivity, strong penetration ability, long signal half-life, and repeatable activation of probes etc. Wang et al. Utilized the water-soluble trianthracene derivative-based nanoparticles (TD NPs), using the delayed ultrasound-excited luminescence imaging (DELI) mode to accurately image brain tumors, subcutaneous tumors, and peritoneal metastases. They also designed an ultrasound-excited molecular luminescent probe (TD-Grz-BHQ) for granzyme B response and achieved an accurate assessment of distal effects of oxaliplatin combined with anti-PDL1 in mice treated with oxaliplatin through the imaging of specific and sensitive detection of granzyme B [50].

#### 4.1.3 Multimodal imaging

In recent years, the multimodal diagnostic imaging modality in which ultrasound is combined with other imaging technologies (e.g. MRI, PAI, PET, etc.) has aroused extensive scientific interest. This modality can accurately collect multidimensional information simultaneously, break through the limitations of a single imaging modality, and enhance the sensitivity and specificity through the complementary advantages between different technologies, which has clinical translational potential in the fields of oncology and cardiovascular diseases [[51], [52], [53]]. Pathak et al. constructed multifunctional contrast agents for both MRI/US imaging. The team coupled MB containing superparamagnetic iron oxide nanoparticles (SPION) with cyclic RGDfK (Arg-Gly-Asp), resulting in RGD-SPION-MB that specifically targets αvβ3 integrin receptors on the neovascular system of mice with breast cancer *in vivo*, which significantly improves the imaging efficiency [54]. Moreover, Sun et al. constructed gas-generating laser-activatable nanorods for contrast enhancement (GLANCE) particles, with the advantages of high biocompatibility and tunable light absorption. GLANCE emits photoacoustic signals laser excitation, and can achieve the glance can emit photoacoustic signals under laser excitation, enabling ultrasound/photo-acoustic multimodal imaging, while GLANCE can reduce the acoustic impedance through the photocatalytic function of gold nanorods and the photolysis of azides to generate nitrogen vesicles to improve the relative lack of contrast in ultrasound imaging [55].

### 4.2 Therapeutic applications

#### 4.2.1 Sono-optical therapy (SOT)

SOT utilizes ultrasound waves to excite biomaterials to emit light or produce other forms of energy conversion to enable precise treatment of specific locations in the body. SOT facilitates the treatment of deep tissues, as well as ensuring precise energy delivery to the target area without causing damage to surrounding healthy tissues [5] **(**Table 2**)**.

**Table 2:** Therapeutic applications of ultrasound-responsive micro-/nanoplatforms for SMT and SOT.

Biomaterials	Material Type	*In Vitro*	*In Vivo*	Administration Route	Effect	US Parameter	Treatment Method	Application	Ref.
cRGD-NBs	Nanobubbles	BMSCs	Mouse calvarial defect model	Local injection into the calvarial defect region	Mechanical effect	3 MHz, 100 mW/cm^2^, 50 % duty cycle	SMT	Promoting osteogenesis of BMSCs and bone formation for fracture healing	[27]
Azo-PEG hydrogel	Mechanophore hydrogel	B16F10, E0771	−	−	ROS	550 kHz, 115 W/cm2	SMT	Noninvasive cancer treatment by generating ROS to kill tumor cells	[60]
PCL/PVDF	Piezoelectric nanofibers	Schwann cells, PC12 cells	Rat sciatic nerve 15 mm defect model	Implanted nerve conduits	Electrical effect; Mechanical effect	1 MHz, 0.5 W/cm^2^, 20 % duty cycle	SMT	Peripheral nerve regeneration by promoting nerve function recovery and axonal maturity	[29]
PTMB	Targeted microbubbles	N2A cells, rat hippocampal neurons	−	−	Mechanical effect, Ca^2^^^+^^ transients	2 MHz	SMT	Low-intensity ultrasound neuromodulation via mechanosensitive ion channels	[141]
FeAlg/PEI/MSCL	Nanogel	Hela, CT26, B16F10, 4 T1 cells	C57BL/6 melanoma model	i.t.	Mechanical effect, Immune activation	6 MHz	SMT + Immunotherapy	Cancer immunotherapy via apoptosis-induced DC maturation and T cell activation	[61]
MBs	Microbubbles	PDAC cell lines (BxPC3/AsPC1/PANC1)	Subcutaneous xenograft tumor model in BALB/c nude mice	i.t.	Mechanical effect	1 MHz, 1.0 W/cm^2^, 50 % duty cycle	SMT	Induces apoptosis in PDAC via Piezo1 activation, providing a non-invasive therapeutic strategy	[30]
mSZ@PDA-NO NPs	Mesoporous silica-based NPs	H22, NIH 3T3, RAW 264.7	Mouse tumor model	i.v.	Photothermal, NO release, Mechanical effect	2 MHz, 2 W/cm^2^	SPT + Gas + SMT	Synergistic tumor ablation via self-propelled motion and NO/PTT effects	[57]
Lipo@IR780/L012	Liposome nanoparticles	HEK cells expressing CheRiff opsins	Thy1-ChR2-YFP transgenic mice	i.v.	ROS, blue light emission	1.5 MHz	Sonogenetic-optogenetic	Noninvasive activation of motor cortex neurons for limb movement control	[25]
FUSION	Nanoparticles	Bone Marrow-Derived Macrophages (BMDMs)	4 T1 tumor-bearing mice	i.v.	ML, R848 release, ·OH-enhanced persistent luminescence	1.5 MHz, 33.9 W/cm^2^, 10 % duty cycle	Immunotherapy with closed-loop control	FUS-triggered macrophage M1 polarization, real-time optical feedback, tumor suppression, and immune memory induction	[58]
MLNTs	Mechanoluminescent nanotransducers	PDMS phantom, Artificial circulatory system	Thy1-ChR2-YFP mice	i.v.	Mechanical effect	0.65–3.5 MHz	Sono-optogenetics	Optogenetic neuromodulation, deep-tissue imaging	[7]
BTB-OCn	Organic mechanoluminescent materials	Crystals, Planar devices, Capsule devices	Subcutaneous implants	−	Mechanical effect	−	US-ML Imaging	Subcutaneous phosphorescence imaging with high SBR for biomedical applications	[56]
Lipo@IR780/L012/CaO_2_	Lipid nanotransducers	Primary neuron culture	Mouse M2 cortex and VTA	Intracranial injection	Mechanical effect, ROS, light emission	1.5 MHz	Sono-optogenetics	Deep brain neuromodulation, activation of ChR2-expressing neurons, behavioral control	[59]

Abbreviations: SMT, Sono-mechano therapy; SOT, Sono-optical therapy; ROS, Reactive oxygen species; i.t., Intra-tumor injection; i.v., Intravenous injection; PTT, Photothermal therapy; Immuno, Immunotherapy; Gas, Gas therapy; SPT, Sono-piezo therapy; NO, Nitric oxide; MSC, Marrow stem cells; US, Ultrasound; ML, Mechanoluminescence.

**Fig. 3:**
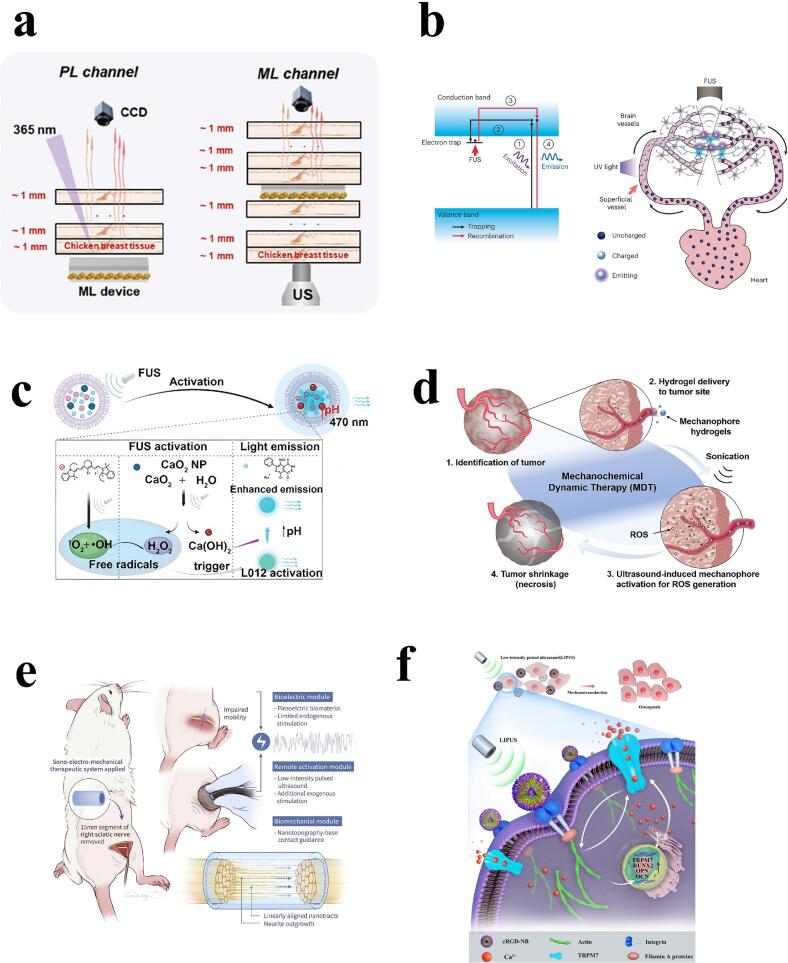
Applications of SOT and SMT. a) Schematic of bio-imaging studies with organic mechanoluminescent materials. Reproduced with permission from ref. [56], Copyright 2024, Wiley. b) Schematic diagram of the mechanism of focused ultrasound stimulation of luminescence and its effects *in vivo*. Reproduced with permission from ref. [7], Copyright 2023, Springer Nature. c) Schematic diagram of the mechanism and effect of ultrasound-triggered liposome mechanoluminescence system for optogenetic stimulation of deep brain tissues. Reproduced with permission from ref. [59], Copyright 2023, American Chemical Society Publications. d) Schematic representation of the mechanism of activation of mechanophores in ultrasound-controlled hydrogels. Reproduced with permission from ref. [60], Copyright 2022, National Academy of Sciences. e) Schematic diagram of the sono-electro-mechanical therapy system. Reproduced with permission from ref. [29], Copyright 2023, Elsevier. f) LIPUScRGD-modified nanobubbles that can promote osteogenesis of bone marrow mesenchymal stem cells (BMSCs). Reproduced with permission from ref. [27], Copyright 2022, Open Access.

Initially, researchers have explored how to utilize organic mechanoluminescent (ML) materials to achieve fluorescence and phosphorescence emission in the red/NIR region under ultrasonic excitation [56] **(**Fig. 3**a)**. This technique not only extends the application range of ML materials, but also significantly improves their penetration capability, making them a powerful tool in bioimaging. For example, by designing specific conjugated backbone stacking patterns and intermolecular interactions, the emission properties of ML materials can be tuned to optimize their performance in subcutaneous imaging [56]. Further studies have shown that ultrasound, as a non-invasive source of mechanical stimulation, can effectively activate ML materials and facilitate their application in biomedical fields [26]. Such materials are not only suitable for imaging, but also for the development of drug delivery systems. In particular, ultrasound-triggered ML materials exhibit unique advantages in controlling the location and timing of release, offering the possibility of personalized therapy.

In the field of tumor therapy, Janus mSZ@PDA-NO nanoparticles have been demonstrated to be a multifunctional platform [57], that is capable of generating persistent near-infrared (NIR) afterglows, photothermal effects, and self-driven motions in the presence of ultrasound waves. These properties help to increase the level of permeability and cellular internalization within the tumor, thereby enhancing antitumor efficacy and reducing side effects. In addition, focused ultrasound (FUS)-responsive mechanoluminescent nanoplatforms demonstrate the ability to engineer control of macrophages [58]. Activation of nanosensors by FUS can deliver light signals in deep tissues *in vivo* [7] **(**Fig. 3**b)**, which in turn precisely modulates macrophage behavior, which opens up new pathways to enhance therapeutic efficacy.

Notably, the ultrasound-triggered liposome mechanoluminescence system can be used as an effective noninvasive tool for optogenetic stimulation of deep brain tissues [59] **(**Fig. 3**c)**. This technique not only improves the depth of light penetration and reduces damage to biological tissues, but also offers new possibilities for future clinical applications, especially in the treatment of neurological disorders. The researchers have developed a liposome-based nanoparticle system [25], which is capable of generating blue light in a liquid environment triggered by ultrasound waves, which in turn can be used to activate neurons expressing photosensitive proteins. In addition to this, melanin nucleus-containing and adriamycin-containing perfluoropentane nanodroplets hold great promise for translational US-chemotherapy-photothermal cancer therapy [32], demonstrating the potential for the application of sound and light therapies in nanodroplet-based clinical medicine.

#### 4.2.2 Sono-mechanical therapy (SMT)

In SMT, inertial cavitation induced by ultrasound can produce significant mechanical effects at specific locations in the body, which can promote mechanochemical phenomena at the biomolecular and cellular levels, and thus aid in the treatment of diseases [5] **(**Table 2**)**. Nano and micron bubbles, as well-established contrast agents for ultrasound imaging, are widely used to enhance the effect of low-frequency ultrasound stimulation due to the large acoustic impedance difference between them and the surrounding medium. When these bubbles oscillate, they convert externally applied acoustic field energy into localized mechanical forces; this secondary mechanical force is strong enough to activate neighboring mechanosensitive ion channels. In combination with ultrasound stimulation techniques, nanobubbles have demonstrated the ability to remotely modulate cellular activity. This nanobubble-assisted ultrasound strategy has the advantage of being non-invasive and has high spatial resolution, even when dealing with deep tissues, making it possible to precisely manipulate cellular activity in a specific area.

In cancer treatment, it has been found that azo mechanophores embedded in hydrogels activated by HIFU can generate free radicals and convert them into ROS to effectively inhibit tumor growth, demonstrating the great potential of this new type of therapy − known as “mechano-chemodynamic therapy” (MDT) [60], which not only achieves effective killing of tumor cells, but also has highly selective and non-invasive characteristics. In addition, the application of targeted Piezo1 microbubbles combined with ultrasound, this type of approach utilizes specifically designed microbubbles, which, when combined with low-frequency ultrasound, are able to activate the mechanosensitive ion channel Piezo1 on the surface of cancer cells, leading to an increase in calcium in-flow, which in turn promotes apoptosis of the cancer cells **(**Fig. 3**d)**. Another study indicated that engineered gas vesicles could be used to enable sonic-controlled mechanotherapy [28]. By combining biomaterials with ultrasound, effective killing of cancer cells can be achieved while stimulating the body's own immune system to fight against tumors.

In the field of neuromodulation, SMT demonstrates dual potential in peripheral nerve injury repair and cancer immunotherapy by integrating the synergistic effects of piezoelectric nanomaterials and low-intensity pulsed ultrasound (LIPUS). Research indicates that piezoelectric nanofiber bundles can generate electrical activity (output voltage reaching tens to hundreds of millivolts) under LIPUS stimulation, activating voltage-gated calcium ion channels (Ca^2+^/CaMKII/cAMP signaling pathway), promoting neural stem cell differentiation and axonal directed growth. Their functional recovery effects in a 15 mm rat sciatic nerve defect model are comparable to those of autologous transplantation [29] **(**Fig. 3**e)**. In addition, combining the remote modulation capability of nanobubbles [61], Wang et al. proposed a sonogenetics-based nanosystem for activating mechanosensitive ion channels to induce apoptosis for safe and modifiable cancer immunotherapy. The system induces the activation of the corresponding T cells to generate an effective anti-tumor immune response. This technology demonstrates the potential for non-invasive external modulation, especially in the field of neuroscience.

In bone regeneration, the combination of ultrasound and nanobubbles has been shown to be effective in promoting cellular activity during bone regeneration and accelerating fracture healing. In 2022, Yao et al. reported that low-intensity pulsed ultrasound (LIPUS) combined with cRGD-modified nanobubbles (cRGD-NBs) was effective in promoting osteogenesis of bone marrow mesenchymal stem cells (BMSCs) [27]**(**Fig. 3**f)**. Actin microfilaments and TRPM7 ion channels played a key role in this process, and their mutual regulation promoted extracellular Ca^2+^ inward flow and enhanced the effect of LIPUS/cRGD-NBs on BMSCs. This study not only provides new ideas for optimizing the treatment of fracture healing, but also demonstrates the great potential of the combination of nanotechnology and ultrasound technology, which lays the foundation for the development of non-invasive treatment methods.

#### 4.2.3 Sono-piezoelectric therapy (SPT)

As an emerging ultrasound-based therapy, SPT has garnered significant interest due to its non-invasive and controllable characteristics. The core of SPT lies in the use of specific piezoelectric materials to generate an endogenous electric field (EnEF) under the action of ultrasound waves, thereby activating biochemical reactions within the cell **(**Table 3**)**. Studies have shown that such electric fields have great potential for application in a variety of scenarios, including activation of cellular biochemical reactions, effective inhibition of tumor growth, promotion of bone regeneration and antimicrobials, and provision of precise treatments of the nervous system.

**Table 3:** Therapeutic applications of SPT and STT.

**Biomaterials**	**Material Type**	***In Vitro***	***In Vivo***	**Administration Route**	**Effect**	**US Parameter**	**Treatment Method**	**Application**	**Ref.**
PGBT	Electrospun membrane	MC3T3-E1 cells	Rat cranial defect	−	Piezoelectricity, ROS	0.1 W/cm^2^	SPT	Bone regeneration and anti-bacterial properties promotion	[67]
Sv-MoS_2_ NF	Piezo-sonosensitizer	4 T1 cells	4 T1 tumor-bearing mice	i.v.	ROS	1.0 MHz, 1.5 W/cm^2^, 50 % duty cycle	SPT	Cancer therapy	[62]
SBN/SNO-PEG NCs	Heterostructured piezoelectric nanocomposites	Hepa 1–6 cells	Hepa 1–6 cells in nude mice	i.v.	ROS	1.0 MHz, 1.2 W/cm^2^, 50 % duty cycle	SPT	Cancer therapy	[65]
HZnO-BPQDs-PEG	Piezoelectric hollow ZnO heterostructures	4 T1 cells	4 T1 tumor model	i.v.	ROS, Zn2^+^ and PO_4_^3^^^−^^ release	1.0 MHz, 1.5 W/cm^2^, 50 %duty cycle	SPT	*In vitro* inhibition of cancer cell activity, *in vivo* suppression of tumor growth, induction of autophagy and ferroptosis in cancer cells	[63]
BTO-OVP	Ferroelectric material	4 T1 cancer cells	4 T1 tumor-bearing Balb/c mice	i.v.	ROS, CO	1 MHz, 1.5 W/cm^2^, 50 % duty cycle	SPT	Tumor therapy via ROS and CO production	[142]
BFO NSs	Piezocatalyst	4 T1 breast cancer cells	4 T1 tumor-bearing Balb/c mice	i.t.	ROS	1 MHz, 1.0 W/cm^2^, 50 % duty cycle	SPT + PMS	Tumor treatment by generating ROS	[64]
CM-PNPs	Piezoelectric nanoparticles	HMC-3 microglia cells, U87 MG glioblastoma cells	−	−	Piezoelectric effect, Microglia polarization, ROS	939 kHz, 1.7 W, 10 % duty cycle	SPT	Inducing microglia polarization to M1 phenotype to enhance antitumor activity	[143]
BTNP-pDA-BNN6	Piezoelectric nanoparticles	Differentiated SH-SY5Y cells	MPTP-induced Parkinson’s mice	i.v.	Current stimulation, Nom BBB opening, dopamine release	1.5 MHz, 462.4 W/cm^2^, 10 % duty cycle	SPT	Alleviates Parkinson’s symptoms, neuroprotection	[144]
KBGO	Nanocomposite hydrogel	BMSCs	Rat calvarial defect model	Injectable implantation into bone defect site	Electrical stimulation, Mechanical reinforcement	1.5 W/cm^2^, 50 % duty cycle	SPT	Accelerated healing of irregular bone defects via calcium influx and PI3K/AKT/ERK pathways	[68]
RP NPs	Red Phosphorus NPs	4 T1 cancer cells, L929	4 T1 tumor-bearing mice	i.v.	Thermal effect, ROS, ATP depletion	1 MHz, 0.5 W/cm^2^, 50 % duty cycle	STT + SDT	Mild sonothermal therapy for deep tumors with HSP inhibition	[70]
HA-NC_Cu	Cu single-atom coordinated nanocubes	MDA-MB-231 breast cancer cells, 4 T1 cells, HUVEC	MDA-MB-231 tumor-bearing mice	i.v.	ROS	1.0 MHz, 1.0 W/cm^2^	STT + CT	Synergistic sonothermal-catalytic therapy for triple-negative breast cancer, inducing apoptosis and ferroptosis	[145]
TiO_2_	Metal oxide (rutile TiO_2_)	S. aureus, E. coli	Rat subcutaneous infection model	−	Thermal effect, ROS, bacterial trapping	1 MHz, 1.2–1.5 W/cm^2^, 50 % duty cycle	STT	Antibacterial therapy for implant-associated infection, enhanced osseointegration	[71]
PINH	Core-shell structured nanomaterial	−	SCC7 tumor − bearing nude mice model	Peritumoral injection	Gas generation, Drug release, Cavitation	40 MHz (imaging), 10 MHz (therapy)	STT	Deep-seated tumor theranostics	[146]
Ti-RP	Metal-Semiconductor	MRSA	MRSA-infected bone	Implanted	Sonothermal, NO	1 MHz, 1.0 W/cm^2^	STT + NO Gas Therapy	Eradicate deep MRSA infection	[72]

Abbreviations: SDT, Sonodynamic therapy; MOF, Metal–organic frameworks; MRSA, Methicillin-resistant *Staphylococcus aureus*; STT, Sonothermal therapy; CT, Catalytic therapy; ATP, Adenosine triphosphate; BBB, Blood-brain barrier; PMS, Peroxymonosulfate.

**Fig. 4:**
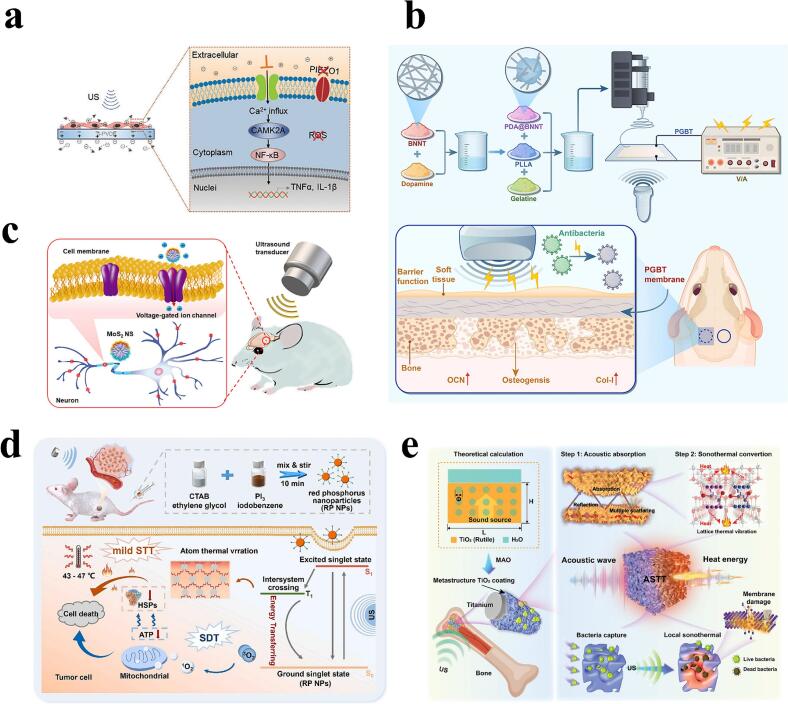
Mechanisms and biological effects of SPT and STT. a) Mechanism diagram of SPT regulation of macrophage polarization. Reproduced with permission from ref. [66], Copyright 2021, Open Access. b) Schematic representation of the osteogenesis mechanism after LIPUS-stimulated application of PGBT membrane. Reproduced with permission from ref. [67], Copyright 2025, Elsevier. c) Schematic representation of a remote and selective method for modulating neuronal activity using piezoelectric molybdenum disulfide NS and ultrasound. Reproduced with permission from ref. [69], Copyright 2023, American Chemical Society. d) Schematic diagram of the synthetic protocol for RP NPs and the combined antitumor mechanism. Reproduced with permission from ref. [70], Copyright 2024, Elsevier. e) Schematic design of TiO_2_*meta*-structural coating and its antimicrobial STT mechanism. Reproduced with permission from ref. [71], Copyright 2023, Wiley.

In cancer therapy, various types of piezoelectric materials have been designed to improve the effectiveness of cancer treatment. For example, molybdenum disulfide (MoS_2_) and its sulfur vacancy-modified version (Sv-MoS_2_ NF), as highly efficient piezoelectric sensitizers [62], are able to significantly enhance the efficiency of electron-hole pair separation under the action of ultrasound waves, promote the generation of ROS, and effectively inhibit the growth of tumors [63,64], as well as displaying good biocompatibility and safety [65]. In addition, SPT can also exert its antitumor effects through other special effects. For example, piezoelectric materials can modulate the polarization state of macrophages, in particular enhancing their M1 (pro-inflammatory) phenotype to improve anti-tumor immune responses [66]. Pro-inflammatory macrophages (M1) play an important role in anti-tumor immunity, and regulating the polarization of pro-inflammatory macrophages is key to immunotherapy. Based on this, Kong et al. used local electrical signals generated by piezoelectric *β*-phase polyvinylidene fluoride (*β*-PVDF) membranes to regulate macrophage polarization, thereby significantly inhibiting tumor cell proliferation [66] **(**Fig. 4**a)**.

In terms of bone regeneration and antimicrobial, it was shown that a novel piezoelectric GBM membrane dispersed by boron nitride nanotubes could can promote *in vivo* osteogenesis with potential for repair of vertical bone defects [67] **(**Fig. 4**b)**. The simultaneous generation of ROS had a significant effect on bacterial membrane rupture and death. This provides a new strategy for bone regeneration and antimicrobial resistance. In addition, hydrogel-based bone repair materials, especially those capable of releasing drugs or promoting bone tissue regeneration in response to changes in mechanical forces, have demonstrated their potential in personalized medicine [68].

For the treatment of neurological disorders, the efficient conversion of ultrasound energy into localized electrical stimulation using piezoelectric molybdenum disulfide nanosheets (MoS_2_ NS) offers the possibility of selectively modulating one or more targeted brain circuits, promising precise stimulation of neurons within the brain [69] **(**Fig. 4**c)**. This approach may be efficacious for treating brain circuit dysfunction and neurological deficits. Specific piezoelectric signals can direct neural stem cells to differentiate along predetermined pathways, providing new therapeutic ideas for nerve damage repair and neurodegenerative diseases [69]. In summary, these materials are not only biocompatible, but also dynamically adjust their physicochemical properties according to external conditions, offering the possibility of future clinical applications.

#### 4.2.4 Sono-thermal therapy (STT)

STT is an advanced ultrasound technology, which facilitates local heating of biological tissues by converting ultrasound energy into thermal energy. In recent years, with the development of nanotechnology and material science, the combination of US and biomaterials with sono-thermal conversion effects has demonstrated the enormous application potential of STT in fields such as antitumor and antibacterial therapy [5] **(**Table 3**)**.

In the domain of tumor treatment, various types of sono-thermogens with different effects have been developed to enhance their antitumor effects. Ma et al. have reported that red phosphorus nanoparticles (RP NPs) act as sono-thermogens. Under the irradiation of low-intensity ultrasound, RP NPs exhibit a significant temperature rise effect, reaching up to 47 °C, enabling effective mild STT [70] **(**Fig. 4**d)**. In this study, STT therapy overcame the limitations of conventional photothermal therapy in deep tumor treatment, laying the foundation for the development of more effective cancer treatments in the future.

In terms of antimicrobial applications, a porous coating based on titanium dioxide (TiO_2_) using the mechanism of STT and its mechanism deserve further exploration [71]. It can effectively absorb acoustic energy when receiving ultrasonic signals and convert it into thermal energy, thereby achieving local high temperatures to kill bacteria **(**Fig. 4**e)**. Guan et al. have found that the coating exerts a significant inhibitory effect on *S. aureus* and *E. coli*, among others. Furthermore, red phosphorus nanoparticles can be put to use in fighting MRSA infection [72]. A heterojunction composite material (Ti-RP) formed by titanium (Ti) and red phosphorus was successfully prepared using chemical vapor deposition. This material exhibited a significantly enhanced sono-thermal effect, capable of raising the temperature by more than 20 °C within 25 min.

Notably, in order to ensure the safety and effectiveness of STT, Guan et al. have also carried out a large number of theoretical calculations and experimental verification. By adjusting the pore size, thickness and porosity of the porous coating, the ultrasound absorption capacity can be effectively controlled, thus improving the effectiveness of STT and ensuring the biocompatibility of the coating [71]. In summary, based on these research findings, STT exhibits great promise as a non-invasive and highly effective treatment method and is likely to become a crucial tool in the treatment of various diseases in the future.

#### 4.2.5 Sonodynamic therapy (SDT) and its synergistic therapy

##### 4.2.5.1 SDT

SDT has been widely studied due to its high tissue penetration capability, excellent therapeutic efficacy, and biological safety. As a novel non-invasive treatment modality, three main factors influence its therapeutic efficacy: US, sonosensitizers, and ROS sources [20]. In recent years, researchers have conducted extensive studies on these three influencing factors in fields such as tumors, infectious diseases, and cardiovascular diseases **(**Table 4**)**.

**Table 4:** Biomedical applications of SDT.

**Biomaterials**	**Material Type**	***In Vitro***	***In Vivo***	**Administration Route**	**Effect**	**US Parameter**	**Treatment Method**	**Application**	**Ref.**
SPNC1	Semiconducting polymer	4 T1 cancer cells	4 T1 tumor mouse mode	i.v.	ROS	50 kHz, 1 W/cm^2^ , 50 % duty cycle	SDT	Enhancing penetration ability SDT for large solid tumor	[73]
PEBVO@PEG NRs	Semiconductor nanorod	4 T1 cells	4 T1 tumor-bearing mouse model	i.v.	ROS	1 MHz, 1.5 W/cm^2^ , 50 % duty cycle	SDT	Photoetching oxygen vacancies to enhance SDT against hypoxic tumor	[147]
AB-MN	TiO_2_ microneedles	NIH3T3	Wound biofilm model	Therapy patch was inserted into the infected site	Cavitation effects; ROS; Low-temperature thermal effects	1 MHz, 50 % duty cycle, 1.5 W/cm^2^	SDT	Eradicate deep-layered wound biofilm	[78]
MIL@Ag-PEG	MOF (Ti)	A549 and MCF-7 cells; *S. aureus*	Tumor-bearing mice; *S. aureus* infection model	i.v.； covered wound with a sterile dressing	ROS	2.0 W/cm^2^	SDT	Deep-seated cancer and bacterial infection	[148]
Pd@Pt-T790	Nanozyme	MRSA	Mice with MRSA infection	i.v.	ROS	1.0 MHz, 0.97 W/cm^2^, 50 % cycle	SDT	Eradicate MRSA-induced myositis	[79]
5-ALA	Organic Sonosensitizers	THP-1 cells	C57BL/6J apoE-/- mice	i.v.	ROS	1.0 MHz, 0.5 W/cm^2^, 10 % duty cycle	SDT	Enhances efferocytosis, cholesterol efflux and anti-inflammatory reactions for atherosclerosis treatment	[81]
CCNU980 NPs	Organic fluorophore	BV2 cells	Myocardial I/R model	Microinjected into the PVN	ROS	1 MHz, 50 % duty cycle； 0.5 W/cm^2^ (*in vitro*), 2.0 W/cm^2^(*in vivo*)	SDT	Protect against myocardial ischemia–reperfusion injury	[84]
BBTD-TPA	Organic sonosensitizers	BV2 cells	MI model	Microinjected into the PVN	ROS	1 MHz, 50 % duty cycle； 0.5 W/cm^2^ (*in vitro*), 2.0 W/cm^2^(*in vivo*)	SDT	Post-myocardial infarction neuromodulation and arrhythmia prevention	[85]
RuB	Supramolecular coordination complexes (SCCs)	BV2 cells	MI model	Microinjected into the PVN	ROS	1 MHz, 50 % duty cycle； 0.5 W/cm^2^ (*in vitro*), 2.0 W/cm^2^(*in vivo*)	SDT	Enhancing autophagy to prevent ventricular arrhythmias post-myocardial infarction	[86]
LFO@GOx	Perovskite Nanoenzyme	4 T1 cancer cells	4 T1 breast cancer model	i.v.	Quadruple enzyme-mimicking activities, ROS	1.0 MHz, 1.2 W/cm^2^ , 50 % duty cycle	EDT	Pyroptosis-dominant breast cancer treatment	[149]
CaF_2_	Injectable nanozyme hydrogel	4 T1 cancer cell, H22 cancer cell	4 T1 breast cancer and H22 hepatic carcinoma models	i.t.	Peroxidase-Mimicking Activity, ROS	1.0 MHz, 1.2 W/cm^2^ , 50 % duty cycle	EDT	Ultrasound-amplified and Ca^2+^-overload-enhanced catalytic tumor nanotherapy	[150]

Abbreviations: I/R, Ischaemia/reperfusion; PVN, Paraventricular nucleus; MI, Myocardial infarction; EDT, Enzyodynamic therapy; TPA, Tissue plasminogen activator.

**Fig. 5:**
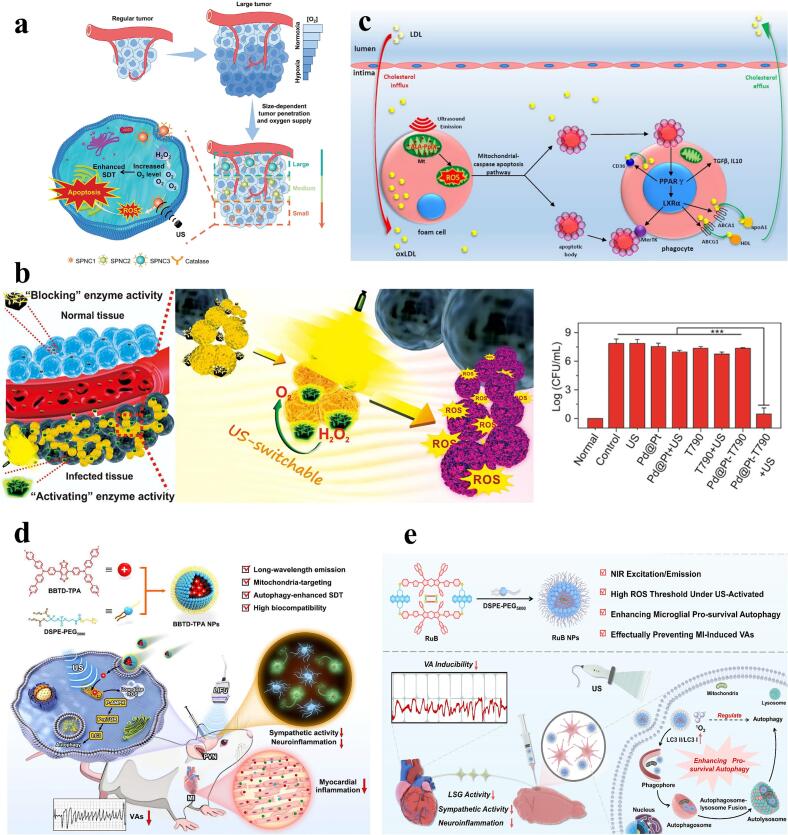
Biomedical applications of SDT in tumors, infections, and cardiovascular diseases. a) Schematic representation of the size-dependent penetration ability of SPNC1 to increase tumor oxygenation during enhanced SDT in large solid tumors. Reprinted with permission from ref. [73]. Open Access. b) Schematic diagram of Pd@Pt-T790 nanoplatform for bacterial infection treatment and its therapeutic effect. Reprinted with permission from ref. [79]. Copyright 2020, American Chemical Society. c) ALA-mediated SDT anti-atherosclerotic mechanism. Reprinted with permission from ref. [81]. Copyright 2018, IVYSPRING. d) Schematic representation of the synthesis process of BBTD-TPA nanoparticles and the mechanism of SDT regulation against ventricular arrhythmias (VAs) after myocardial infarction (MI). Reprinted with permission from ref. [85]. Copyright 2025, IVYSPRING. e) Schematic diagram of the RuB functioning as a sonosensitizer to regulate ROS levels and mitigate MI-induced VAs. Reprinted with permission from ref. [86]. Copyright 2025, Wiley.

Cancer therapy represents a predominant application of SDT. However, the tumor microenvironment (TME) often exhibits properties such as hypopermeability and hypoxia, which inevitably limit the therapeutic efficacy of SDT [73]. A range of strategies have been developed to improve the hypoxic TME, including the delivery of exogenous O_2_ [74], reducing O_2_ consumption by tumor cells [75], and generating O_2_ *in situ* within the tumor [73]. Wang et al. developed a small-sized sonosensitizer, SPNC1 (35 nm), which has two main advantages. Firstly, its small size allows for greater penetration capacity. Secondly, it is covalently linked to catalase, which reacts with excess hydrogen peroxide in the TME and generates O_2_ in situ. This has been shown to improve the TME and thus enhance the efficacy of SDT [73] (Fig. 5a). In addition, to overcome the limitations of oxygen-dependent SDT (type II) in hypoxic TME, an increasing number of studies have begun to focus on the development of oxygen-independent SDT (type I) or type I/II sonosensitizers, which generate higher levels of ROS and help overcome the limitations of SDT in hypoxic TME [76,77]. As exemplified by Liu e, a mitochondria-targeted 2D nanoscale covalent organic framework (nCOF), Td-Pc, achieved prolonged circulation, high tumor accumulation, and potent type- I/II SDT effects. This nanoplatform generated substantial ROS in tumor tissues, thereby causing pyroptosis of immunogenic cancer cells [76].

SDT has also been extensively studied for the treatment of infectious diseases. Biofilm infections of wounds are characterized by their inherent resistance to antibiotics, and photodynamic therapy (PDT) has been successfully used to treat biofilm-induced wound infections, but the weak penetration of light limits its application to deep biofilm infections. US can achieve higher tissue penetration, and therefore SDT for deep biofilm infections appears to have more potential for development. Nevertheless, the delivery of the sonosensitizer to the site of infection remains a significant challenge. Ouyang et al. applied hyaluronic acid microneedle (MN) patches loaded with sonosensitizer to wounds, accelerated MN dissolution by ultrasonic cavitation, which led to rapid dispersion of the loaded sonosensitizer into the biofilm, and utilized MN penetration of the skin barrier and biofilm to achieve rapid debridement of deep tissue infections and promote wound healing [78]. Furthermore, it has been demonstrated that hypoxia frequently occurs at the site of infection. Sun et al. proposed an US-switchable nano-enzyme system (Pd@Pt-T790), where the modification of T790 on Pd@Pt significantly blocked the catalase-like activity of Pd@Pt, but its activity was effectively restored under ultrasound irradiation to catalyze the decomposition of endogenous H_2_O_2_ into O_2_. This dual modality of 'blocking and activating' the enzyme activity has the potential to alleviate the hypoxic microenvironment at the infection site, while concurrently reducing the potential toxicity and side effects of the nano-enzymes on normal biological tissues [79] **(**Fig. 5**b)**.

In recent years, the applications of SDT in the field of cardiovascular diseases have also attracted considerable attention. Tian et al. found that 5-aminolevulinic acid (ALA)-mediated SDT could induce foam cell apoptosis through the mitochondrial-caspase pathway, leading to atherosclerosis treatment [80]. Their subsequent in-depth study revealed that ALA-mediated SDT may enhance cholesterol efflux and efferocytosis through activation of the PPARγ-LXRα-ABCA1/ABCG1 pathway, inducing an anti-inflammatory response and ultimately ameliorating atherosclerosis [81] **(**Fig. 5**c)**. Recent studies have also found that macrophages are an important component of atherosclerotic plaques, and promoting the apoptosis of diseased macrophages can reduce the occurrence of early atherosclerosis [82]. Therefore, SDT for targeting diseased macrophages has also been widely studied. Cao et al. found that hyaluronic acid (HA) is an effective targeting molecule that selectively binds to the CD44 molecule. The expression of the CD44 protein is upregulated in activated macrophages, accompanied by an increase in affinity for HA. Therefore, they utilized HA-modified CuS/TiO_2_ heterostructure nanosheets (HA-HNS) to develop a targeted SDT for treating early-stage atherosclerosis by targeting macrophages within plaques [83]. Moreover, recent studies have applied SDT to neuromodulation and cardiac arrhythmia prevention. Hu and Pang et al. designed BBTD-TPA and supramolecule RuB nanoparticles for SDT to promote microglial autophagy and inhibit sympathetic neuroinflammation via the ROS-AMPK-mTOR pathway, thereby reducing ventricular arrhythmias after myocardial infarction [[84], [85], [86]] **(**Fig. 5**d, e)**.

##### 4.2.5.2 SDT combined with chemodynamic therapy (CDT)

CDT can cause oxidative damage to tumor cells by converting endogenous H_2_O_2_ to hydroxyl radicals (·OH) in an acidic environment via the Fenton or Fenton-like reaction, catalyzed by transition metal containing biomaterials [87]. This treatment can achieve tumor therapy without external stimuli, relying only on endogenous tumor chemical energy **(**Table 5**)**. Nevertheless, the efficacy of CDT is limited by endogenous H_2_O_2_ and insufficient acidity[88]. It has been found that the local turbulence of ultrasonic shock waves can significantly enhance the Fenton response [88,89]. Therefore, it is hypothesized that the combination of CDT and SDT may result in a substantial increase in the efficiency of ROS generation, thereby enhancing the therapeutic efficacy. Lin et al. developed engineered FePS_3_-PEG NSs as sonosensitizer/Fenton nanocatalysts for dual nanodynamic tumor therapy. On the one hand, the material can be used for SDT of tumors by generating ROS via electron transfer reaction under the action of ultrasound; on the other hand, Fe^2+^ contained in the material can catalyze the overexpression of H_2_O_2_ in the acidic TME to generate highly toxic ·OH via Fenton reaction for CDT of tumors. In addition to this, Fe^3+^ generated by oxidation of Fe^2+^ can efficiently consume glutathione (GSH), thus significantly increasing the oxidative damage effect of SDT and CDT, and further enhancing the therapeutic effect of tumor treatment [88].

**Table 5:** Synergistic strategies of SDT and other therapies.

**Biomaterials**	**Material Type**	***In Vitro***	***In Vivo***	**Administration Route**	**Effect**	**US Parameter**	**Treatment Method**	**Application**	**Ref.**
FePS_3_-PEG NSs	Semiconductor materials	4 T1 cancer cells	BALB/c mice bearing 4 T1 tumors	i.v.	ROS	1.0 MHz, 0.5 W, 50 % duty cycle	SDT + CDT	Dual nanodynamic tumor therapy	[88]
Cu_2−x_O − BTO NCs	Piezoelectric materials	4 T1 cancer cells	4 T1 tumor bearing BALB/c mice	i.v.	ROS	1.0 MHz, 1.0 W/cm^2^ , 50 % duty cycle	SDT + CDT	Improvement in cancer therapy using piezoelectric heterostructures	[87]
OA/Ce6	Carrier-free nanosensitizer	PC9 cell, 4 T1 cell	4 T1 tumor bearing BALB/c mice	i.v.	ROS, Antineoplastic OA	1 MHz, 100 % duty cycle, 0.1 W/cm^2^ (*in vitro*), 1.0 W/cm^2^(*in vivo*)	SDT + PDT + Chemo	Synergistic chemo/sono-photodynamic therapy for cancer treatment.	[90]
TiH_1.924_-PVP nanodots	Metal hydride nanomaterials	4 T1 tumor cells	4 T1 tumor-bearing mice	i.v.	ROS, Photothermal effect	40 kHz, 3 W/cm^2^, 1 min per cycle, 20 cycles	SDT + PTT	Metal hydride nanomaterials as physical stimuli-triggered nanoagents for cancer treatment.	[151]
Pt-CuS-P-TAPP	Hollow Pt-CuS Janus	CT26 cancer cells	CT26 tumor-bearing mice	i.v.	ROS, Catalase-like activity; Photothermal effect	1.0 MHz, 60 % duty cycle, 0.5 W/cm^2^ (*in vitro*), 1.0 W/cm^2^(*in vivo*)	SDT + PTT	PTT and catalysis-enhanced SDT could realize complete tumor eradication	[91]
UCNPs-C_3_N_4_-Ce6	Upconversion nanomaterial	B16-F0 cells	B16-F0 tumor-bearing mice	i.t.	ROS, Photothermal effect	–	SDT + PDT + PTT	Noninvasive deep-site tumor therapy via ideal autophagy mechanisms	[92]
MagO_2_MB-RB-Gem	Microbubbles	BxPC-3 and Mia-PaCa-2 cells	Xenograft ectopic BxPC-3 tumors in SCID mice	i.v.	ROS, Gemcitabine	–	SDT + Chemo	Magnetic microbubble mediated chemo-SDT for pancreatic cancer	[94]
MOF@CPT_2_-Azo	MOF	4 T1 breast cancer cells	4 T1 tumor-bearing mice	i.v.	ROS, Camptothecin	1.0 W/cm^2^	SDT + Chemo	Sequential SDT and tumor hypoxia-activated sono-chemotherapy	[95]
HMME/R837@Lip	Nano-liposomes	4 T1 cancer cell; CT26 cancer-cell	4 T1 tumor-bearing mice; CT26 tumor-bearing mice	i.v.	ROS, Anti-PD-L1 checkpoint blockade	1.0 MHz, 1.5 W/cm^2^, 50 % duty cycle	SDT + Immuno	Noninvasive tumors-therapeutic modality with immunotherapy	[152]
SPN_Ab_	Semiconducting polymer nanobodies	4 T1 cells	4 T1-tumor-bearing BALB/c mice	i.v.	ROS, Anti-CTLA4 checkpoint blockade	1 MHz, 1.5 W/cm^2^, 50 % duty cycle	SDT + Immuno	Activatable cancer sono-immunotherapy	[97]
GCZ@M	ZIF-8 coated with tumor cell membrane	4 T1 cells	4 T1 tumor-bearing mice	i.v.	ROS, NO	1.0 MHz, 1.0 W/cm^2^ (*in vitro*)；1.5 W/cm^2^ (*in vivo*)	SDT + Gas	High-performance cancer therapy	[98]
Pt-Bi_2_S_3_	Metal–semiconductor heterostructure nanocomposites	4 T1 cells	4 T1 tumor-bearing mice	i.v.	ROS, H_2_	1.0 MHz, 50 % duty cycle, 1.5 W/cm^2^	SDT + Gas	Sonocatalysis-mediated cancer therapy	[99]
P-BTO	Barium titanate nanoparticle	4 T1 cells	4 T1 tumor-bearing mouse	i.v.	ROS, O_2_	1.0 MHz, 1.0 W/cm^2^ , 50 % duty cycle	SDT + Gas	Highly efficient hypoxic tumor therapy	[100]
M/LPV/O_2_	Biomimetic sonosensitizer	Cal-27 cells	Orthotopic OSCC mice	i.v.	ROS, O_2_	1.0 MHz, 0.35 W/cm^2^ (*in vitro*); 2.0 W/cm^2^ (*in vivo*)	SDT + Gas	Orthotopic oral cancer therapy	[101]
[Ru(bpy)_3_]^2+^	Polypyridinal metal complexes	4 T1 tumor cells	4 T1 tumor-bearing mice	i.t.	ROS	3.0 MHz, 0–0.3 W/cm^2^	SDT + NADH consumption	Metal complexes in the noninvasive sonotherapy of cancer	[153]
ZrO_2−x_@Pt/AIPH	ZrO_2−x_-based nanoplatform	4 T1 tumor cells	4 T1 tumor-bearing mice	i.v.	ROS, Free alkyl radicals	1.0 MHz, 1.0 W/cm^2^, 50 % duty cycle	SDT + TDT	Sonodynamic-thermodynamic therapy for tumor eradication and metastasis inhibition	[102]
TiO_2_@Pt/GOx	Inorganic sonosensitizer	4 T1 cells	4 T1 tumor-bearing mice	i.t.	ROS, Glucose depletion	1.0 MHz, 1.0 W/cm^2^ , 50 % duty cycle	SDT + starvation therapy	Cancer treatment	[103]
C-ZnO	MOF	4 T1 tumor cells	4 T1 tumor-bearing mice	i.v.	ROS, Cavitation effects	1 MHz, 1.5 W/cm^2^, 50 % duty cycle	SDT + mechanical therapy	Enhancement of ROS production and US-induced mechanical effects for cancer therapy	[104]
P/M@CasMTH1	MOF	A549 cells	A549 tumor-bearing mice	i.v.	ROS, Genome Editing	1.0 MHz, 1.5 W/cm^2^, 50 % duty cycle	SDT + Genome editing	Cancer treatment	[105]
g-ZnN_4_-MoS_2_	Porphyrin-like Zn single-atom catalysts	MC3T3-E1 osteoblasts	Osteomyelitis model	Marrow cavity injection	ROS, Zn^2+^	1 MHz, 50 % duty cycle, 1.5 W/cm^2^	SDT + ion therapy	Treatment of MRSA-infected osteomyelitis	[106]

Abbreviations: CDT, Chemodynamic therapy; PDT, Photodynamic therapy; Chemo, Chemotherapy; COF, Covalent organic framework; Immuno, Immunotherapy; NADH, 1,4-dihydronicotinamide adenine dinucleotide; TDT, Thermodynamic therapy.

##### 4.2.5.3 SDT combined with phototherapy (PT)

**Fig. 6:**
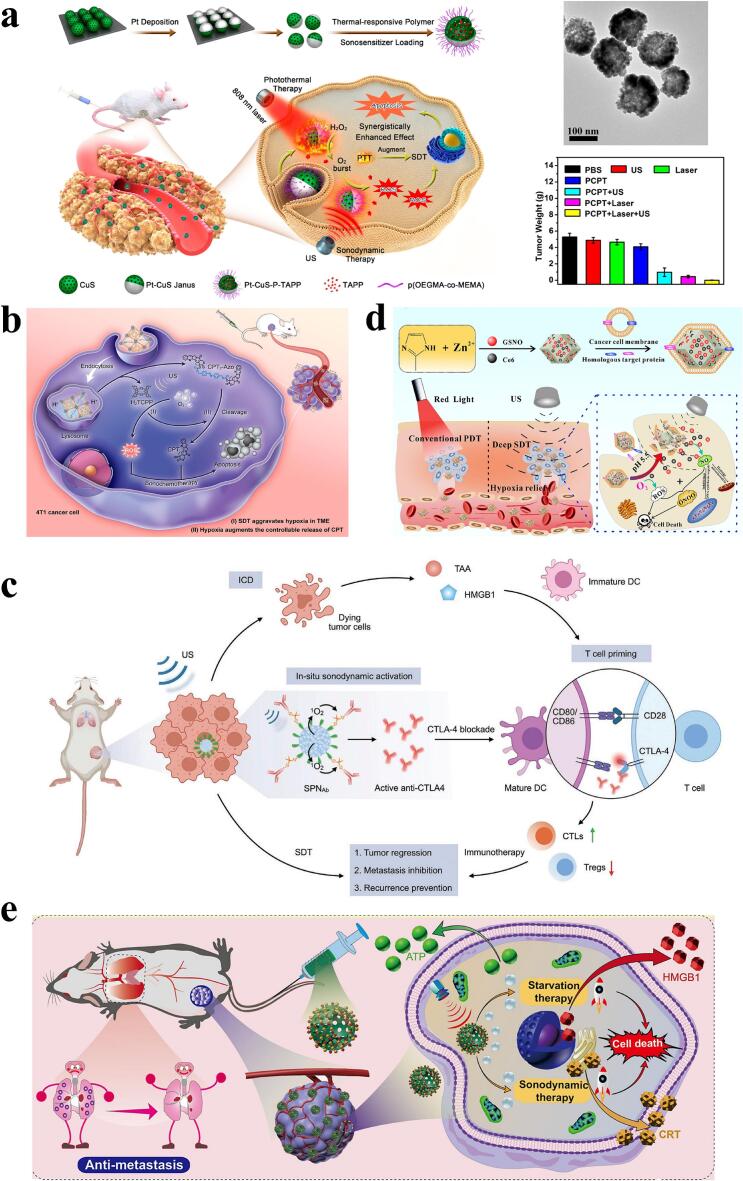
Biomedical applications of SDT in combination with other therapies. a) Anti-tumor mechanism of action, TEM images and tumor therapeutic effect of Pt-CuS-P-TAPP. Reprinted with permission from ref. [91]. Copyright 2019, American Chemical Society. b) Mechanisms of US-driven sono-chemotherapeutic treatment of MOF@CPT_2_-Azo. Reprinted with permission from ref. [95]. Copyright 2022, American Chemical Society. c) Schematic of SPN_Ab_-mediated activatable sono-immunotherapy. Reprinted with permission from ref. [97]. Copyright 2022, Wiley. d) Therapeutic mechanism of GCZ@M nanoparticle gas therapy combined with SDT. Reprinted with permission from ref. [98]. Copyright 2019, Elsevier. e) Schematic of SDT combined with starvation therapy. Reprinted with permission from ref. [103]. Copyright 2022, American Chemical Society.

PT, which mainly consists of photodynamic therapy (PDT) and photothermal therapy (PTT), has been widely used in therapeutic studies of oncology and infectious diseases as a non-invasive, low-toxicity treatment [20]. However, poor light penetration ability, potential phototoxicity and other undesirable factors have limited its further development. With the continuous development of nanomedicine technology and SDT, many sensitizers are suitable for both PT and SDT, the research on synergistic treatment of SDT and PT has been well developed, which can be achieved by synergistic therapeutic effects, and can be exerted with fewer sensitizers/ultrasound/light doses than monotherapy. This results in better therapeutic results [20,90] **(**Table 5**)**. In addition, the photothermal effect generated by PTT can enhance intra-tumor blood flow and improve tumor oxygenation, thereby improving the hypoxic TME and enhancing the therapeutic effect of SDT. The Pt-CuS-P-TAPP nanomaterials developed by Liang et al. not only combine the therapeutic effects of SDT and PTT, but also have nano-enzymatic activity of the precious metal Pt that can catalyze the decomposition of endogenous H_2_O_2_ to produce O_2_ to improve the hypoxic TME, and the thermal effect generated by the laser irradiation also accelerates the catalytic activity of the Pt, which further enhances the therapeutic effect of SDT [91] **(**Fig. 6**a)**. In addition, the research on triple combination therapy of SDT with PDT and PTT is gradually increasing, which can significantly improve the treatment effect, and is being applied in the research of various tumor treatments such as melanoma and breast cancer [20,92].

##### 4.2.5.4 SDT combined with chemotherapy

Chemotherapy is a commonly used clinical treatment for tumors, in which cytotoxic chemotherapeutic drugs are used to achieve therapeutic effects by blocking the growth and proliferation of cancer cells. However, most chemotherapeutic drugs have poor tumor-targeting ability, high systemic toxicity and serious side effects, which greatly hinder their development [93]. Many studies have developed a variety of targeted drug delivery systems, which have greatly improved the tumor targeting and therapeutic efficacy of drugs **(**Table 5**)**. For example, Beguin et al. incorporated magnetic materials into MBs while loading 5-fluorouracil (chemotherapy drug) and Rose-Bengal (sonosensitizer), thereby synthesizing MagO_2_MBs with dual targeting functions, namely US-responsive drug delivery and magnetic-responsive targeting. By using a specific device that combines magnetic array with US transducer to activate the biomaterial, targeted delivery of the chemotherapy drug and sonosensitizer was achieved, thereby combining SDT with chemotherapy to treat pancreatic cancer [94]. In addition, by making full use of the hypoxic characteristics of the TME, controlled release of chemotherapeutic drugs can also be achieved, thus reducing their systemic toxicity and improving therapeutic effects. Zhuang et al. designed a sequential US/hypoxia-activated nanomedicine (MOF@CPT_2_-Azo) by initially synthesizing the hypoxia-activated Azo bond-containing camptothecin (CPT) prodrug (CPT_2_-Azo) into a metal–organic framework (MOF) integrating a sonosensitizer. Upon entry of the drug into the tumor hypoxic microenvironment, the loaded non-toxic CPT_2_-Azo prodrug was released from the MOF, and the US-activated cytotoxic ROS were generated via SDT, which consumed a large amount of oxygen exacerbating the microenvironmental hypoxia. Hypoxia in turn promotes the activation of CPT_2_-Azo, enabling the controlled release of cytotoxic chemotherapeutic agents (CPT) and compensating for the defective efficacy of SDT due to hypoxia [95] **(**Fig. 6**b)**.

##### 4.2.5.5 SDT combined with immunotherapy

Tumor immunotherapy, which mainly includes the fields of chimeric antigen receptor T-cell therapy (CAR-T), immune checkpoint inhibitors, cancer vaccines, and other cellular therapies, has been a very hot research direction for tumor treatment in recent years. However, most cancer immunotherapies may have safety issues, targeting effects, off-target side effects, autoimmune reactions, and other shortcomings that limit their further clinical translation [3]. Intriguingly, a number of preclinical studies have found that SDT induces tumor cell death by generating cytotoxic ROS and that tumor cell debris can act as tumor-associated antigens to trigger anti-tumor immune responses [96]. Inspired by the intrinsic therapeutic effect of SDT and the potential property of inducing an immune response, synergistic treatment of tumors with SDT and immunotherapies seems to achieve better therapeutic outcomes **(**Table 5**)**. Zeng et al. constructed nanoparticles SPN_Ab_ with relatively low binding affinity for CTLA-4 by binding anti-CTLA-4 antibodies to polymer nanoparticles via ^1^O_2_-cleavable junctions. Upon ultrasound irradiation, SPN_Ab_ produces ^1^O_2_, which not only induces SDT effect to induce immunogenic cell death, but also releases anti-CTLA-4 antibodies and triggers the checkpoint blockade [97] **(**Fig. 6**c)**. This ^1^O_2_-responsive nanotherapeutic platform enables immune checkpoint blockade therapy precise and controllable, greatly reducing the risk of off-targeting.

##### 4.2.5.6 SDT combined with gas therapy

Gas therapy, a method of relieving disease through the use of exogenous gases, has attracted the attention of many researchers as a new type of ‘green’ therapy [3]. Commonly used therapeutic gases are nitric oxide (NO), oxygen (O_2_), hydrogen (H_2_) and hydrogen sulphide (H_2_S) [[98], [99], [100], [101]]. However, the current clinical application of direct inhalation and oral routes of administration is only effective for surface lesions in the lungs and the gastrointestinal tract, but not for deep inflammation or tumors, which cannot enrich enough gases to achieve satisfactory therapeutic effects [99]. Therefore, the construction of nanoparticles with unique biological characteristics, which can release or generate therapeutic gases at the lesion site through external stimulation, has effectively solved the above-mentioned challenges. US, as a method with high tissue penetration capability, has demonstrated its immense potential in this application [3] **(**Table 5**)**. For instance, An et al. constructed a multifunctional biomimetic nanoplatform, GCZ@M, which has homologous targeting properties due to the encapsulation of the tumor cell membrane for effective aggregation at the lesion site. When triggered by US, the release of NO from the encapsulated nitrosoglutathione (GSNO) and the production of ROS from chlorin e6 (Ce6) reacted to produce highly reactive peroxynitrite (ONOO^–^) molecules and other reactive nitrogen species (RNS), which are more lethal oxidants, to enhance the therapeutic effect of the tumor [98] **(**Fig. 6**d)**.

##### 4.2.5.7 SDT combined with other therapies

In addition to the above combined treatment strategies, SDT can be used in synergy with other therapies to enhance the therapeutic effect, mainly including thermodynamic therapy (TDT), starvation therapy, mechanotherapy, genome editing and ion therapy [[102], [103], [104], [105], [106]] **(**Table 5**)**. Depletion of nutrients in the tumor cells would hinder the stable supply of cellular energy thus inducing tumor cell starvation and greatly enhancing the therapeutic effect of SDT. Zhao et al. developed TiO_2_@Pt/GO_x_ nanoparticles, in which glucose oxidase (GO_x_) can efficiently catalyze the oxidation of glucose to gluconic acid and hydrogen peroxide (H_2_O_2_) to deplete glucose-induced cellular starvation. At the same time, Pt nanoenzymes can catalyze the decomposition of excess H_2_O_2_ produced by glucose oxidation and generate a large amount of O_2_ to alleviate tumor hypoxia. In combination with the inorganic sonosensitizer TiO_2_ generating ROS, it promotes oxidative damage and energy depletion of tumor cells [103] **(**Fig. 6**e)**. In addition, Feng et al. designed g-ZnN_4_, a sonosensitizer with dual functions of SDT antibacterial and osteogenic promotion. Not only can it generate ROS under ultrasound stimulation to achieve antibacterial effects, but Zn single atoms immobilized in g-ZnN_4_ can be released steadily in the form of Zn^2+^ within a safe concentration, realizing the great osteoinductive ability of such a sonosensitizer. This SDT-combined ion therapy treatment strategy is used to treat osteomyelitis caused by MRSA infections [106].

#### 4.2.6 Ultrasound-responsive drug delivery

Ultrasound-responsive delivery systems harness ultrasound to precisely control drug release, enhance targeting, and improve delivery efficiency. These systems are proving to be versatile tools in various therapeutic areas, mainly including tumor, cardiovascular diseases, and neurological disorders **(**Table 6**)**.

**Table 6:** Applications of sonogenetics and US-induced gene and drug delivery.

**Biomaterials**	**Material Type**	***In Vitro***	***In Vivo***	**Administration Route**	**Effect**	**US Parameter**	**Treatment Method**	**Application**	**Ref.**
Adenovirus-associated virus carriers	Viral vector	SH-SY5Y neuroblastoma cells	Parkinson's disease mice	Local viral injection	Improved dopaminergic neuronal viability	Low frequency (0.5 MHz), Burst duration (3 s), and low energy (0.5 MPa)	Sonogenetics	Treatment of neurodegenerative diseases	[118]
pBV220 Plasmid/E. coli MG1655	Engineered bacteria	Breast cancer cells	4 T1 tumor-bearing mice	i.v.	Upregulation of IFN-γ, Regulation of Immune Cell	Sound pressure (3.52 Mpa), Frequency (960 Hz), Pulse period (150 ms)	Sonogenetics	Induction of cancer cell apoptosis	[119]
PCF-MBs	Microbubble	HT-29 colon cancer cells	HT-29 cancer bearing Balb/c nude mice	i.v.	CPT; FUDR; ROS	1.0 MHz, 1 W/cm^2^, 50 % duty cycle	US-induced drug delivery + PDT	Overcoming drug resistance of colorectal cancer	[107]
MDNPs	Biomimetic nanosonosensitizer system	Human GBM U87 cells	GBM bearing Balb/c nude mice	–	DOX; ROS	1 MHz, 2 W/cm^2^	US-induced drug delivery + PDT	Deep-seated and drug-resistant tumors.	[108]
IRMB-OxLipo	Microbubble-liposome complex	Panc-01 3D spheroids	BxPC-3 human xenograft murine model of pancreatic cancer	i.v.	Irinotecan; Oxaliplatin	1 MHz, 3.5 W/cm^2^, 30 % duty cycle	US-induced drug delivery	Improving the overall effectiveness of this drug combination	[37]
iTSL-DOX	Liposome	–	TNBC (MDA-MB-231) tumor-bearing mice	i.v.	DOX	1.4 MHz, 10–20 W, 25 % duty cycle	MRI-guided FUS +chemo	MRI assists the application of FUS for precise drug release and therapy	[154]
DMC@P-Cs	Cerasomal nano-modulator	CT26 cells	CT26 tumor-bearing mice	i.v.	ROS; DMC	1 MHz, 1 W/cm^2^	HIFU + immuno	Synergistical SDT-immunotherapy for enhanced colorectal cancer therapy	[155]
PEOz-Lip@R837	pH-responsive liposome	Panc02 cells	Panc02 tumors −bearing C57BL/6 mice	i.v.	Toll-like receptor agonist (R837)	1.0 MHz, 0.5 W/cm^2^, 20 % duty cycle	US improved adjuvant delivery	UMC- and pH- sensitive immune adjuvant delivery-based treatment	[156]
CNDs	Chitosan/perfluorohexane nanodroplet	LNCaP cells	–	–	DKK-2 pDNA	1 MHz, 1 W/cm^2^, 50 % duty cycle	US-induced gene delivery	Achieving spatiotemporally controlled US-mediated gene delivery	[33]
CpMBs	Cationic porphyrin microbubble	Human breast cancer MCF-7 cells	MCF-7 tumor xenograft-bearing female BALB/c nude mouse model	i.v.	siRNA; ROS	1.03 MHz, 50 % duty cycle, 1 W/cm^2^ (*in vitro*)；1.03 MHz, 50 % duty cycle, 2 W/cm^2^ (*in vivo*)	US-induced gene delivery + PDT	Excellent therapeutic effect for estrogen-dependent ER_+_ breast cancer	[31]
PAP@Lipid	Nanodroplet	4 T1 adherent cells	4 T1 breast cancer model	i.t.	ARTA; PTX; ROS	1 MHz, 1 W/cm^2^, 50 % duty cycle	US-induced drug delivery	Therapy for refractory cancers	[157]
hDox-NB	Nanobubble	N1-S1 rat hepatoma cell line	HCC rat model	i.v.	DOX	3 MHz, 2.2 W/cm^2^, 10 % duty cycle	US-induced drug delivery	Improving patient outcomes and decrease off-target side effects	[158]
NO-ELIPs	Echogenic liposome	Flow circuit model	Atherosclerotic miniature swine model	Intraarterial administration via catheterization	NO	2.3 MHz	FUS + Gas	Obviating the need for long-term	[36]
RGDS-targeted microbubbles	Microbubble	–	Thrombotic rabbit model	i.v.	Urokinase	–	US-induced drug delivery	Achieving a complete recanalization of the femoral artery	[159]
MMB-SiO_2_-tPA	Microbubble	–	Femoral vein thrombi-bearing mouse model	i.v.	tPA	10–900 kHz	US-induced drug delivery + MDT	Time-critical thrombolytic therapy	[160]
H_2_S-MBs	Microbubble	Artery thrombus model	Left iliac artery thrombi	i.v.	H_2_S	1.9–2 MHz	FUS + Gas	Thrombosis treatment	[161]
PNBs-siNox2	Biomimetic nanobubble	HUVECs	ApoE-/- mice	i.v.	Nox2 siRNA	1.0 MHz, 0.5 MPa, 20 % duty cycle	US-induced gene delivery	Atherosclerosis treatment	[162]
Cationic microbubbles	Microbubble	–	Acute myocardial infarction rats	i.v.	MSC; PDGF-BB	1.0 MHz, 2.0 W/cm^2^, 20 % duty cycle	US-induced cell/drug delivery	Improving AMI therapy	[163]
rtPA-loaded MB	Microbubble	Artery thrombus model	Coronary no-reflow mongrel dogs	Intraarterial administration via catheterization	rtPA	–	US-induced drug delivery	Adjuvant therapy for reperfusion therapy	[164]
DREADD-AVV	Viral vector	–	C57BL/6J mice	i.v.	Clozapine-N-oxide	1.5 MHz, 0.42 MPa	US-induced gene delivery + MDT	Treatment of neurodegenerative and neuropsychiatric diseases	[111]
Propofol-loaded nanoemulsions	Nanoemulsion	–	PTX-induced acute epilepsy rat	–	Propofol	1.0 MHz	MRI-guided FUS-induced delivery	Non-invasive targeted transcranial neuromodulation therapy	[109]
Definity MBCA	Nanodroplet	–	SD rats	i.v.	Pentobarbital	580 kHz	US-induced drug delivery	Applied to local neural regulation	[112]
DNA/PEI complex-loaded cationic microbubbles	Microbubble	MRC-5 cells	BALB/c mice	i.m.	HBsAg	0.5 MHz	US-induced gene delivery	Improving transfection efficiency and immunogenicity	[165]
LNPs	Nanobubble	–	Mouse cervical heart transplantation model	i.v.	Antagomir-155	1.0 MHz, 360 kPa, 20 % duty cycle	US-induced gene delivery	Improving heart transplant rejection	[166]
Cationic porphyrin microbubbles	Microbubble	Neonatal rat cardiomyocytes	Hypertension-induced cardiac hypertrophic	i.v.	Anti-miR-23a	1.3 MHz	US-induced gene delivery	Treatment of cardiac hypertrophy and heart failure	[167]
PFH-PLGA-NH2-NB	Nanobubble	Isolated porcine skin model	Nude mice	Transdermal	Celecoxib	650 kHz, 2.0 W, 50 % duty cycle	US-induced drug delivery	Treatment of Achilles tendon adhesion	[113]
iELPs	Liposome	HUVECs, MH7As, RAW 264.7	CIA mouse model	i.v.	MTX	1.0 MHz, 0.2 MPa	US-induced drug delivery	Rheumatoid arthritis treatment	[114]

Abbreviations: ROS, Reactive oxygen species; CPT, Camptothecin; FUDR, Floxuridine; GBM, Glioblastoma; DOX, Doxorubicin; TNBC, Triple-negative breast cancer; HCC, Hepatocellular carcinoma; MDT, Magneto-dynamic therapy; ARTA, All-trans retinoic acid; tPA, Tissue-type plasminogen activator; PTX, Paclitaxel; PDGF, Platelet-derived growth factor; i.v., Intravenous injection; i.t., Intratumoral injection; i.m., Intramuscular injection; CIA, Collagen-induced arthritis; MTX, Methotrexate.

##### 4.2.6.1 Biomaterials with intrinsic ultrasound-responsive drug delivery capabilities

**Fig. 7:**
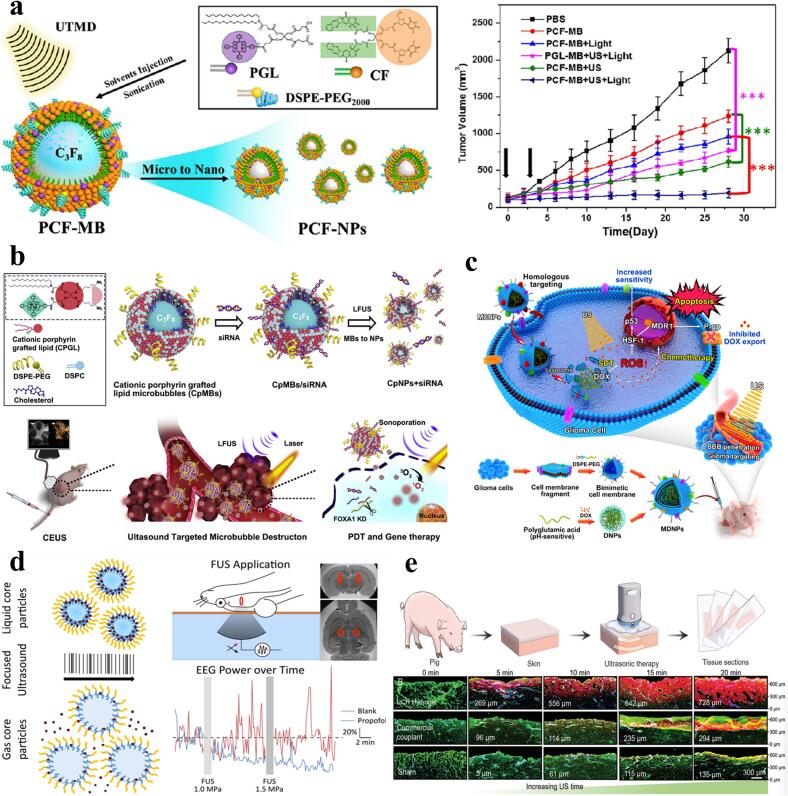
Therapeutic applications of ultrasound-responsive drug delivery. a) Synthesis and conversion of PCF-MB to PCF-NB process; tumor growth in nude mice 0–30 days after treatment in each group. Reprinted with permission from ref. [107], Copyright 2018, American Chemical Society. b) Schematic illustration of the strategy of loading porphyrin microbubbles, ultrasound-assisted PDT, and FOXA1 KD by siRNA. Reprinted with permission from ref. [31], Copyright 2018, Elsevier. c) Mechanism and synthesis process of MDNPs for GBM treatment. Reprinted with permission from ref. [108], Copyright 2022, American Chemical Society. d) Principle of use of isopropanol nanoemulsion; localization maps of rats with seizures and the effect of emulsion treatment under ultrasound. Reprinted with permission from ref. [109], Copyright 2017, American Chemical Society. e) Mechanism and immunofluorescence staining image of Ultrasound Nanobubble Coupling Agent for effective noninvasive deep-layer drug delivery, Reprinted with permission from ref. [113], Open Access.

This strategy focuses on carriers designed to release their payload or facilitate delivery when exposed to ultrasound. Microbubbles (MBs), nanobubbles (NBs), liposomes, and lipid nanoparticles (LNPs) are frequently used. One compelling example comes from Chen et al., who developed high-drug-loading porphyrin/camptothecin-fluorouracil tri-micelles (PCF-MBs). When combined with ultrasound and laser irradiation, these micelles achieved a remarkable 90 % inhibition rate of colorectal cancer, demonstrating the potential for precise drug delivery and effective tumor suppression [107] **(**Fig. 7**a)**. Furthermore, another innovative approach involves PNPs nanoparticles. To treat atherosclerosis, Hu et al. created platelet membrane-coated biomimetic nanobubbles (PNBs-siNox2) loaded with Nox2 siRNA. These nanobubbles leveraged the natural targeting ability of platelets to accumulate at atherosclerotic plaques, and with ultrasound, they facilitated targeted antioxidant therapy to stabilize the plaques [31] **(**Fig. 7**b)**.

##### 4.2.6.2 Ultrasound for blood–brain barrier opening in central nervous system drug delivery

The blood–brain barrier (BBB) is a significant obstacle for delivering drugs to the brain. Ultrasound can temporarily and reversibly open the BBB, allowing therapeutic agents to reach the central nervous system. In 2023, Chen et al. addressed glioblastoma treatment by developing biomimetic nano-sonosensitizing systems (MDNPs). These involved doxorubicin (DOX)-loaded nanoparticles encapsulated within glial partial cell membranes. Under ultrasound, the MDNPs successfully crossed the BBB, were taken up by GBM cells, and demonstrated enhanced anti-tumor effects with no significant cardiotoxicity [108] **(**Fig. 7**c)**. Moreover, in a different approach, Airan et al. utilized an isopropanol nanoemulsion for neuromodulation. This nanoemulsion of the small molecule anesthetic propofol, when activated by ultrasound, effectively suppressed epileptic seizures without causing any damage to the brain parenchyma, highlighting its potential for targeted neuromodulation [109] **(**Fig. 7**d)**. Currently, the primary way to open BBB is FUS. When selecting parameters, it is important to maximize the opening of the BBB while avoiding bleeding or exudation. For the common parameters for FUS to open BBB, they are 0.5–1.5 MHz in frequency, 0.3–0.6 MPa in intensity, 0.5 %-2% in duty cycle, and the most suitable pulse width length is 10 ms [[108], [109], [110], [111], [112]]. However, these parameter ranges are mostly derived from animal experiments, thus individual differences need to be taken into account during clinical trials.

##### 4.2.6.3 Ultrasound-mediated transdermal drug delivery

Ultrasound can increase the permeability of biological barriers, allowing drugs to penetrate deeper into tissues or be delivered non-invasively through the skin. For instance, in the realm of non-invasive transdermal drug delivery, ultrasound enables the delivery of coupling agents, achieving drug penetration depths significantly greater than traditional methods. This opens up possibilities for administering various medications without injections [113] **(**Fig. 7**e)**. Additionally, ultrasound plays a crucial role in enhancing drug delivery for specific conditions like rheumatoid arthritis. It can be used to load targeting peptides to improve the permeability of methotrexate (MTX), a common drug for rheumatoid arthritis, thereby enhancing its therapeutic effect in the affected joints [114].

However, ultrasound still faces many challenges in clinical translation that require in-depth research. Most importantly are its safety and controllability: ultrasound effects may damage normal cells due to cavitation, potentially even damaging the blood–brain barrier, and the cytotoxicity of the drug carrier itself may also harm the human body. Therefore, the biosafety of ultrasound-responsive carrier drugs and the control of ultrasound parameters still need further discussion. In the future, packaging strategies and ultrasound design need to be further optimized to increase drug accumulation in target tissues and reduce distribution in normal tissues, truly achieving controllable and safe ultrasound-responsive carrier drug delivery, and promoting the widespread clinical adoption of this method.

#### 4.2.7 Sonogenetics

Sonogenetics employs genetic engineering to produce ultrasound-sensitive proteins in target cells and then employs non-invasive ultrasound to induce specific life activities **(**Table 6**)**. Based on different biological effects, ultrasound-sensitive proteins can be divided into mechanosensitive ion channels induced by mechanical effects, such as transient receptor potential (TRP) channels, Prestin variants (N7T, N308S), etc., and thermosensitive ion channels activated by thermal effects, such as temperature-sensitive repressor (TSR), TRP channels, and heat-shock proteins (HSP), etc. [115]. Compared to previous optogenetics and nuclear genetics, Sonogenetics offers more clinical translational advantages due to its excellent tissue penetration depth, precise cellular targeting, high spatial and temporal resolution, and non-invasiveness. Currently, sonogenetics has shown great clinical potential in the fields of deep brain neuromodulation, tumor therapy, cardiovascular diseases, ophthalmology, stem cell therapy, etc. [116,117]. Fan et al. demonstrated that Prestin protein expressed by a single injection of adeno-associated virus was significantly persistent in dopaminergic neurons in the substantia nigra of Parkinson's disease (PD) mice, and that repeated low-frequency stimulation with transcranial focused ultrasound significantly ameliorated PD symptoms [118]. In addition, Chen's team constructed ultrasound-responsive Escherichia coli MG1655, genetically engineered bacteria that respond to the thermal effects of focused ultrasound, contribute to targeting 4 T1 tumors to release IFN-γ and modulating the activation and polarization of macrophages and T lymphocytes so as to induce apoptosis in cancer cells [119].

### 4.3 Integrated diagnosis and treatment application

Recently, the research of ultrasound diagnostic and therapeutic integration platforms by integrating diagnostic and therapeutic ultrasound technologies has become increasingly popular, and many therapeutic and diagnostic biomaterials have been developed in the field of ultrasound-responsive biomaterials for building ultrasound combined with biomaterials diagnostic and therapeutic integration platforms **(**Table 7**)**. Utilizing imparting imaging properties to therapeutic biomaterials or loading therapeutic drugs into ultrasound imaging contrast agents, ultrasound-responsive biomaterials have both diagnostic imaging and therapeutic capabilities, which can enable long-term monitoring of therapeutic efficacy for use in guiding further treatment of disease.

**Table 7:** Ultrasound-associated with responsive biomaterials for integrated diagnostic and therapeutic applications.

**Biomaterials**	**Material Type**	***In Vitro***	***In Vivo***	**Administration Route**	**Treatment US Parameter**	**Diagnostic Method**	**Treatment Method**	**Application**	**Ref.**
p–n-CD@CCM	NIR phosphorescent carbon dot	143B cells	143B tumor-bearing mouse	i.v.	50 kHz, 3.0 W/cm^2^	NIR imaging	SDT	NIR imaging-guided SDT for tumor therapy	[120]
1-NPs	Nanosensitizers	4 T1 cells	4 T1 tumor-bearing mouse	i.v.	2.0 W/cm^2^ , 50 % duty, 1.0 MHz	NIR FL and MR bimodality imaging	SDT + PDT	Bimodal FL and MR imaging-guided SDT/PDT for breast and brain tumors	[168]
H-TiO_2_/C-PEG	Composite nanosheet	4 T1 cells	4 T1 tumor-bearing mouse	i.v.	0.6 W/cm^2^, 1 MHz, 50 % duty	PAI	SDT + CDT + PTT	Multifunctional diagnostic and therapeutic strategy for tumor therapy	[169]
CR-PEG-GBP	Croconaine dye (CR-1)-based theranostic agent	HepG2, Huh7 and LO2 cells	Mice bearing HepG2 tumors	i.v.	1 MHz, 1.61 W/cm^2^	NIR-II fluorescence imaging; PAI	SDT + PTT	HCC theranostic strategy	[170]
PFP–HMME@PLGA/MnFe_2_O_4_–Ram NPs	Ferrite-loaded multifunctional nanoparticles	RAECs	Advanced rabbit atherosclerotic plaque models	i.v.	1.5 W/cm^2^, 1 MHz	MRI/PA/US multimodal imaging	SDT	Early diagnosis, real-time assessment, and effective treatment of atherosclerotic plaque neovascularization	[123]
α-Fe_2_O_3_@Pt	Inorganic Sonosensitizers	4 T1 cells	4 T1 tumor-bearing mouse	i.v.	1 MHz, 1.0 W/cm^2^ (*in vitro*), 2.0 W/cm^2^(*in vivo*)	US imaging	SDT	Effective tumor inhibition with imaging guidance	[171]
hmSiCREKA-RB-PFH	Hollow mesoporous silica	Artificial thrombus	Rat/pig thrombus model	i.v.	1.0 MHz, 1.5 W/cm^2^	US imaging	SDT + Mechanical thrombolysis	Timely monitoring and efficient thrombolysis in thrombi of tPA resistance	[9]
IR780-NDs	Liposomes	4 T1 cancer cells	4 T1 tumor-bearing mice	i.v.	2.4 W/cm^2^	FL/PA/US imaging	SDT	Theranostics nanoplatform for cancer therapy	[121]
SNAPs	Sonoafterglow nanoparticles	4 T1 cells	4 T1 tumor-bearing mice	i.v.	2.0 W/cm^2^	Molecular sono-afterglow imaging	SDT	Sonoafterglow-guided treatment of breast cancer	[47]
Lip-AIPH	Liposomes	MCF-7 cells	MCF-7 tumor xenograft	i.v.	1.0 MHz, 2.5 W/cm^2^	US imaging	SDT + Gas Therapy	US imaging-guided hypoxic tumor therapy	[122]
PGNAs	Nanoagents	CT26 and FTC133 cells	CT26 tumor-bearing mouse	i.v.	30 kHz, 1.5 W/cm^2^	US imaging	SDT + PTT	Long-term ultrasonic imaging-guided combined therapy of tumors	[8]
TLNs-1@C/R and TLNs-2@C/R	Twins-like nanodrugs	Hepa1-6 cell	Hep1-6 tumor-bearing mice	i.v.	2 MHz, 2.0 W/cm^2^, 20 % duty cycle	US imaging	SDT + Immuno	Ultrasound imaging and triggerable penetrative drug delivery	[172]
Mag-Tar-DL NBs	Nanobubbles	RAW264.7, HUVEC	LSS models	i.v.	1 W/cm^2^, 1 MHz, 50 % duty cycle	US imaging	Drug delivery (R406)	Imaging-guided atherosclerosis treatment	[124]
PFP/PFB/DOX-PPEHD	Nanoprobe	–	Nude mice bearing xenografts of C6 glioma	i.v.	1 MHz, 50 % duty cycle, acoustic pressure 2.0 MPa	US/Fluorescence imaging	Drug delivery (DOX)	Ultrasound imaging and drug delivery against cancer	[125]
D-vPCs-O_2_	Nanodroplets	4 T1 cells	4 T1 tumor-bearing mice	i.v.	–	US imaging	Chemo-hyperthermia therapy	Imaging-guided drug and oxygen delivery	[126]

Abbreviations: NIR, Near-infrared; FL, Fluorescence; MR, Magnetic resonance; PAI, Photoacoustic imaging; LSS, Low shear stress.

**Fig. 8:**
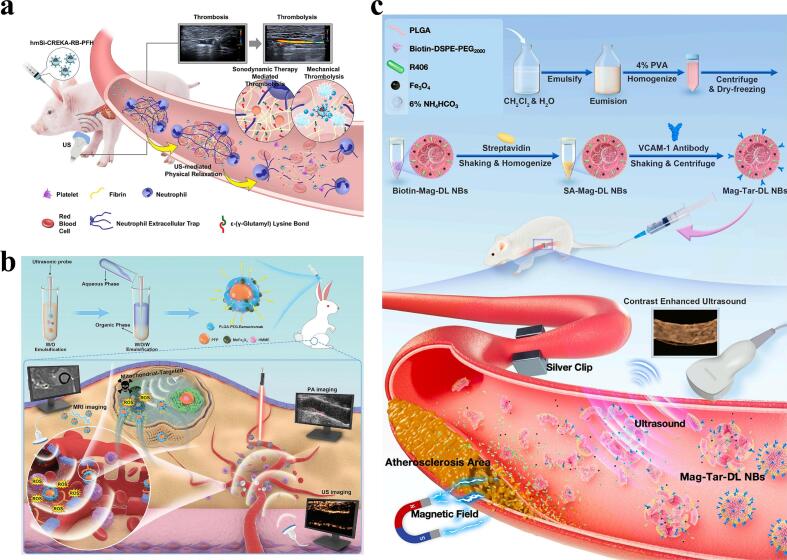
The diagnostic and therapeutic integrated platforms of ultrasound-responsive biomaterials. a) Schematic diagram of the diagnosis and treatment integrated platform of non-invasive real-time ultrasound imaging and efficient thrombolytic. Reprinted with permission from ref. [9]. Open Access. b) Schematic diagram of PFP-HMME@PLGA/MnFe2O4-Ram nanoplatform imaging-guided treatment. Reprinted with permission from ref. [123]. Open Access. c) Schematic diagram of the synthesis process of Mag-Tar-DL NB and its potential mechanism for treating atherosclerosis. Reprinted with permission from ref. [124]. Copyright 2024, Elsevier.

First, by imparting imaging properties to therapeutic biomaterials, it is possible to monitor the distribution of biomaterials in the target area in real-time, thereby determining the ideal treatment window and evaluating the efficacy of treatment [120]. Combining sonosensitizers for SDT with ultrasound contrast agents such as MBs, NBs, and nanodroplets enables ultrasound contrast agent-enhanced imaging-guided SDT. Related integrated diagnostic and therapeutic micro-/nanoplatforms have been extensively studied in fields such as oncology and cardiovascular disease [9,121,122]. For example, Lin et al. found that neutrophil extracellular traps (NETs), ε-(γ-glutamyl) lysine isopeptide bonds, may play a key role in tissue plasminogen activator (tPA)-resistance through thrombus studies in stroke patients. Based on this research foundation, they designed a multifunctional therapeutic diagnostic platform that combines SDT and mechanical thrombolysis with real-time US imaging. The system generates large amounts of ROS through the activation of the sensitizer Rose Bengal (RB) to disrupt NETs and isopeptide bonds; the bursting action of perfluorohexane (PFH) assists thrombolysis through mechanical effects; and the microbubbles generated by PFH act as an ultrasound contrast agent for real-time ultrasound imaging to guide the therapeutic process [9] **(**Fig. 8**a)**. In addition, sonosensitizers can be combined with MRI nanoprobes, near-infrared (NIR) fluorescent groups, etc., to achieve MRI-guided or NIR-guided SDT [120,123]. For example, a NIR phosphorescent carbon dots (CD) therapeutic diagnostic platform p–n-CD @ CCM designed by Geng et al. can be used for NIR imaging-guided SDT tumor therapy. This nanoplatform possesses NIR imaging capabilities, excellent biocompatibility, and efficient ultrasound-triggered ROS generation capabilities. More importantly, by encapsulating the tumor cell membrane (CCM), this biomaterial exhibits high tumor-targeting capability. Therefore, NIR imaging-guided precise SDT therapy enables this nanoplatform to achieve complete clearance of solid tumors with a single injection and a single ultrasound irradiation [120].

In addition, combining sonosensitizers with multifunctional imaging agent, multi-modal imaging-guided SDT can be achieved, compensating for the shortcomings of single-modal imaging, thereby better observing the condition of the lesion area and the distribution of therapeutic biomaterials, and providing timely guidance for treatment [123]. For example, Yao et al. constructed PFP-HMME@PLGA/MnFe_2_O_4_-Ram NPs to achieve MRI and PAI through its loaded manganese ferrite (MnFe_2_O_4_), PFP can be vaporized into gas microbubbles under ultrasound irradiation by acoustic droplet vaporization (ADV) and used for ultrasound enhanced imaging. This MRI/PA/US multimodal imaging tool enables quantitative assessment of atherosclerotic plaque composition, along with plaque neovascularization. In addition, its loaded hematoporphyrin monomethyl ether (HMME) can induce apoptosis of neovascular endothelial cells and inhibit intra-plaque hemorrhage and inflammation through SDT, to achieve the therapeutic effect of stabilizing plaques. The simultaneously loaded Ram, an FDA-approved monoclonal antibody, can bind to vascular endothelial growth factor receptor-2 (VEGFR-2) and inactivate the VEGF-mediated downstream signaling pathway, thereby inhibiting plaque neovascularization and achieving therapeutic effects. This therapeutic diagnostic platform ultimately achieves effective and complete inhibition of plaque neovascularization through multimodal imaging-guided SDT, thereby inhibiting intra-plaque hemorrhage and inflammation, and ultimately stabilizing the plaque [123] **(**Fig. 8**b)**.

Furthermore, MBs, NBs, nanodroplets, and other ultrasound contrast agents can also be used to load other therapeutic drugs such as chemotherapy drugs, anti-inflammatory drugs, and anti-oxidative stress drugs. At the same time, ultrasound contrast agents can be used to enhance imaging and observe the distribution of drugs in the target area, thereby establishing an integrated diagnosis and treatment platform based on ultrasound contrast agents. Related micro-/nanoplatforms have been widely studied in fields such as cancer and cardiovascular disease [[124], [125], [126]]. For example, Lin et al. designed an US imaging-guided drug delivery system with dual targeting properties of magnetism and antibody for the delivery of spleen tyrosine kinase (SYK) inhibitor-R406 to significantly inhibit the inflammatory response and halt the progression of atherosclerosis. The therapeutic diagnostic nanoprobe was endowed with magnetic property by Fe_3_O_4_ and antibody targeting capability by VCAM-1 antibody. Through the dual action of external magnetic field and targeting antibody, the drug-carrying NBs could be effectively aggregated in the region of vascular inflammatory plaques. With diagnostic parameters of ultrasound irradiation, enhanced ultrasound imaging provides better detection of atherosclerotic plaques and drug distribution information; with therapeutic parameters of ultrasound irradiation, NBs are disrupted and R406 is subsequently released in-situ, which inhibits inflammatory response in the target area and prevents atherosclerotic disease progression [124] **(**Fig. 8**c)**.

## 5 Clinical translation

### 5.1 Diagnostic clinical applications

With the increasing demand for early and accurate diagnosis of diseases, ultrasound contrast agents have aroused great interest in scientific research, and various types of new contrast agents have been developed and introduced into clinical trials one after another **(**Table 8**)**. As introduced in the preceding section, the most mature development at the clinical translation level is the microbubble contrast agent, which has now been approved for clinical imaging of the heart and liver [41]. For example, Definity, from Lantheus Medical Imaging, is approved for clinical applications in abdominal and other whole-body imaging. At the same time, Sonazoid offers excellent advantages in diagnosing liver cancer [3,127]. The right heart contrast agent ASI-02 has been reported to be effective in aiding the diagnosis of right-to-left shunts caused by patent foramen ovale failure (PFO) or pulmonary arteriovenous malformation (PAVM), and is now approved for use in Canada, with FDA approval for Phase Ⅲ clinical trials in the United States. Although nanobubbles have the potential to replace microbubble contrast agents, their biosafety has only been validated in mouse models, and their safety and efficacy in humans need to be further investigated. On balance, the novel diagnostic imaging technology of ultrasound combined with biomaterials still faces a series of opportunities and challenges. The low resolution of conventional ultrasound imaging to achieve contrast agent nanoscale imaging urgently requires the development of high-resolution imaging techniques. The biosafety and pharmacokinetic properties of contrast agents still need to be explored in extensive clinical trials [3].

**Table 8:** Clinical translations of ultrasound-responsive bioactive platforms.

Year	Sample size	Subject type	Application	Effectiveness	Safety	Ref.
2017	24(Breast cancer)	Patients with breast cancer and ovarian cancer	Microbubble contrast-enhanced imaging	Significant enhancement of the imaging signal	Good tolerance	[173]
	21(Ovarian cancer)					
2017	24	Patients with prostate cancer	Microbubble contrast-enhanced imaging	Improved detection rates and imaging resolution	Good security	[174]
2018	2105	Patients with breast cancer	Identification of benign and malignant tumors	Improved diagnostic sensitivity for malignant tumors	Good security	[175]
2018	209	Patients with breast cancer	Diagnostic tumor staging	Improving diagnostic accuracy in tumor staging	Good security	[176]
2015	10	Patients with liver neoplasm	Release of ThermoDox®	36.4 % tumor lesion glycolysis decrease	No side effects related to FUS	[128]
2021	18	Patients with unresectable pancreatic ductal adenocarcinoma	Release of ThermoDox®	–	–	[130]
2015	16	Patients with peripheral artery disease	5-ALA-mediated SDT	Long-term efficacy of decline in plaques and vascular stenosis	Good safety without obvious side effects	[135]
2018	32	Patients withsymptomatic femoropopliteal peripheral artery disease	SDT	Significant decrease in target-to-background ratio	No significant differences	[134]
2018	160	Patients with essential tremor, no response to drug	MRgFUS	Significant improvement of the	Dizziness, nausea, vomiting, abnormal sensations and unsteady gait	[177]
				Clinical Rating Scale for tremor		
2020	6	Patients with recurrent glioblastoma	Microbubbles; NaviFUS	–	–	[110]
2023	17	Patients with recurrent glioblastoma	Release of PTX	3.7 times concentration increase	Transient headache	[178]
2024	3	Patients with mild cognitive impairment or mild Alzheimer's dementia	Release of Aducalimumab	Significant amyloid protein decrease	Transient headache	[132]

Abbreviations: FUS, Focus ultrasound surgery; SDT, Sonodynamic therapy; 5-ALA, 5-Aminolevulinic acid; MRgFUS, Magnetic resonance-guided focused ultrasound surgery.

### 5.2 Therapeutic clinical applications

Apart from the trials focused on diagnosis, ultrasound therapy is currently being utilized with increasing frequency in clinical practice. Meanwhile, ultrasound-responsive drug delivery, as an emerging technology with significant advantages, has exhibited greater potential for clinical translation, with many clinical trials conducted for different diseases **(**Table 8**)**.

#### 5.2.1 Clinical applications of ultrasound-responsive drug delivery

In tumor chemotherapy, conventional chemotherapy is difficult to penetrate the tumor stroma, such as the most common chemotherapeutic agent adriamycin, making treatment less effective. In contrast, in the TARDOX Phase I clinical trial, 10 patients with liver tumors were treated with ultrasound-delivered drug therapy, i.e., FUS-mediated release of adriamycin from the heat-sensitive liposome ThermoDox®. The results demonstrated this ultrasound-delivered chemotherapy achieved a 36.4 % reduction in total lesion glycolysis in the targeted tumors without significant adverse effects [128]. TARDOX is the first clinical trial to use ultrasound to triggered thermosensitive liposomes for targeted loading drug delivery, but was suspended at the time of the Phase III trial due to limited effect on liver tumors. This result may have been due to uneven temperature distribution within the tumor, with some temperature zones exceeding the phase transition temperature of ThermoDox® and affecting drug release [129]. In order to further evaluate the effectiveness of ThermoDox®, a similar clinical trial, PANDOX, for unresectable pancreatic ductal adenocarcinoma (PDAC) was conducted in 2021, and the results of the trial have not yet been announced [130].

In neurological disorders of the brain, it is more for the safety and efficacy of drug penetration into the blood–brain barrier appeared different ultrasound devices, launched clinical trials from different angles. For example, the ExAblate Neuro 4000 system, which is used for the treatment of drug-refractory idiopathic tremor, can target and monitor MRgFUS with sub-millimeter precision through MRI and MRI thermography, and utilize FUS to perform targeted thermal ablation of the ventral intermediate nucleus of the thalamus (VIM) to suppress tremor of the responding arm within 3 months, which not only suppresses tremor of the responding arm within 3 months, but also reduces tremor of the responding arm within 3 months. Suppression of tremor in the countermeasure arm resulted in not only a 40–81.3 % reduction in tremor scores within 3 months, but also a 38–50 % improvement in tremor scores at 3 years [131]. In another clinical trial, in three patients with mild cognitive impairment or mild Alzheimer's disease dementia who met the indications for aducanumab treatment, monthly aducanumab infusions and the use of focused ultrasound to open the blood–brain barrier over a 6-month period significantly reduced amyloid levels in the participants (by 48 %, 49 %, and 63 %, respectively), without any decline in cognitive function, providing new ideas for Alzheimer's disease treatment [132].

#### 5.2.2 Clinical trials of SDT

Up to date, there have been clinical trials conducted related to SDT for the treatment of tumors, thrombosis, and atherosclerosis. In a newly published Phase I clinical study in 2025, three newly diagnosed glioblastoma patients received 5-aminolevulinic acid (5-ALA) combined with low-intensity non-targeted ultrasound therapy in the hemisphere before undergoing cytoreductive surgery, reflecting that sonodynamic therapy can promote tumor cell death without damaging brain tissue and may achieve wider spread, such as tumor treatment throughout the entire hemisphere of the brain [133].

Furthermore, unlike heavy therapeutic instruments and expensive MRI chambers, lighter and less expensive SDT devices are also undergoing clinical trials. For example, NaviFUS, a neuronavigation-guided focused ultrasound system, uses a neuronavigation system to pinpoint the location of the tumor and select the appropriate region of interest (ROI), and then uses a hand-held probe to precisely direct the focused ultrasound energy to the ROI area. In combination with microbubbles, it temporarily opens the BBB. Currently, NaviFUS is undergoing a clinical trial to evaluate its safety and feasibility in the treatment of patients with recurrent GBM [110], and its therapeutic role in intreating other neurological disorders, such as Parkinson's disease and Alzheimer's disease, may be explored in the future.

In terms of vascular embolic disease and atherosclerosis, SDT presents satisfactory therapeutic efficacy. For example, in the SMART-PAD clinical trial, patients with symptomatic peripopliteal arterial disease (PAD) in the femoropopliteal artery were treated with SDT, and a reduction in target area/background ratio (TBR) of 0.53 in the most diseased segment (MDS) of the femoropopliteal artery (FPA) in the SDT group could be observed within 30 days [134]. Moreover, it is evident that SDT could achieve a significant reduction in plaque neovascularization and a significant reduction in arterial inflammation by phase I trials of atorvastatin in combination with SDT, and phase II clinical trials of sodium silica-mediated SDT [134,135].

In general, ultrasound-based therapy is consistent with the prevailing trend of non-invasive and precision medicine, and has demonstrated favorable outcomes in clinical trials. Consequently, it is anticipated that the forthcoming expansion of clinical trials will encompass a broader scope of disease treatment and expedite the progression of phase III and IV clinical trials. This will facilitate the elucidation of the characteristics and advantages of diverse ultrasound-based therapy. Meanwhile, the therapeutic modalities, ultrasound-responsive materials and instrumentation need improvement, so as to further ensure the safety, effectiveness and feasibility of ultrasound-responsive drug delivery in clinical translation.

## 6 Challenges and improvement strategies

Despite the considerable potential of ultrasound-responsive micro-/nanoplatforms in the domains of clinical diagnosis and treatment, there are still some significant issues and challenges that hinder its further application. The following discussion will explore these issues and propose feasible improvement strategies.

### 6.1 Further development of ultrasound instruments

In recent years, materials science and nanomedicine have been developing rapidly, but the development of ultrasound-responsive micro-/nanoplatforms also depends on the development of ultrasound instruments. However, the advancement of corresponding ultrasound instruments lags far behind the development of materials science. Currently, most of the ultrasound devices used in related research are clinical ultrasound therapeutic instruments or clinical diagnostic imaging ultrasound, which means that there is no specialized equipment designed for this specific application, which greatly hampers the clinical translation of this emerging technology. Concurrently, diagnostic imaging ultrasound devices and therapeutic ultrasound devices are frequently distinct systems, which often leads to inaccurate localization and treatment of deep diseased tissues, potentially leading to off-target effects. Therefore, an integrated diagnostic and therapeutic ultrasound device that incorporates an ultrasound imaging ‘navigation system’ and a therapeutic ultrasound system can be designed to better facilitate the clinical application of this technology. In addition, this technology can be combined with other newly developed ultrasound technologies, such as wearable ultrasound devices and artificial intelligence ultrasound diagnostic technology [2,136,137], to broaden the scope of applications and satisfy more clinical requirements.

### 6.2 Optimization and standardization of ultrasound parameters

Although current clinical studies demonstrated the effectiveness and biocompatibility of ultrasound-based diagnosis and treatment, there is a lack of standardized protocols for the determination of ultrasound parameters including ultrasound intensity, center frequency, duty cycle, etc. Experiments have relied on literature search and parameter gradient exploration to determine the ultrasound parameters to be used. However, the parameters used in animal experiments are often difficult to apply directly to clinical patients. Therefore, determining standardized protocols for ultrasound parameters is also a great challenge in advancing its clinical translation. One of the reasons why ultrasound parameters are difficult to determine may be that the specific mechanism of action of ultrasound-responsive biomaterials is not yet fully understood. Therefore, a deeper understanding of the specific mechanism of action of ultrasound may better help us to identify relevant parameters for ultrasound applications.

### 6.3 Improving synthesis strategies and refining formulations

The majority of extant research on ultrasound-responsive micro-/nanoplatforms is in the preclinical stage, so researchers focus on the performance of the materials themselves, the effectiveness of the diagnostic treatment, and the biosafety, but not on the cost and process of the synthesis and production of the materials. However, if relevant technologies are to be applied in clinical settings, they must have stable and excellent yields and be economically acceptable to patients. Therefore, the synthesis strategies and formulations should be refined to ensure large-scale, high-standard and reproducible production of biomaterials suitable for clinical patients.

### 6.4 Optimizing the route of delivery of ultrasound-responsive biomaterials

Various biological barriers in the human body and disease microenvironment may prevent drug accumulation in the target therapeutic area, such as the blood–brain barrier and the tumor microenvironment barrier. In some preclinical studies, more invasive modes of drug delivery, such as stereotactic brain injections, intra-tumor injections, etc., may be chosen to maximize the therapeutic effect. Although these modes of delivery may allow for idealization of the therapeutic effect and are suitable for preclinical research operations. However, in the process of clinical translation and promotion of related technologies, the high operational difficulty and low patient acceptance of these more invasive drug delivery methods make the clinical translation of related diagnostic and therapeutic technologies very difficult. Therefore, future research should also concentrate on the development of appropriate, non-invasive or minimally invasive drug delivery methods.

### 6.5 Expanding the biomedical applications of ultrasound-responsive platforms

Whilst researches based on ultrasound-responsive platforms are currently making significant progress in the diagnosis and treatment of malignant tumors, its research applications in other disease areas have yet to be further developed. There are also current studies of its application to the diagnosis and treatment of infectious, cardiovascular, neurological and metabolic diseases, but more comprehensive research is still needed in these areas compared to the great results in malignant tumors. Due to the unique properties of the combination of ultrasound and responsive biomaterials, it is hoped that more research will be conducted in the future to expand the range of biomedical applications to better meet the clinical requirements and alleviate the pain of patients suffering from various diseases.

## 7 Conclusion

Ultrasound is a non-invasive, safety-assured method of energy transmission characterized by high tissue penetration depth and non-ionizing radiation. The diagnosis and therapeutic platform that combines ultrasound as a trigger with responsive biomaterials offers a highly promising approach for medical advancement. By employing various synthesis and material modification techniques to impart diagnostic or therapeutic properties to different biomaterials, this approach enhances their tissue-targeting specificity while ensuring their biocompatibility and biosafety, thereby minimizing drug-related adverse reactions. The current review primarily introduces the diagnostic imaging applications, therapeutic applications, and integrated diagnostic and therapeutic applications of ultrasound-combined biomaterial systems. This approach provides a novel perspective on the development of clinical diagnostic and therapeutic technologies. Nevertheless, the relatively delayed development of this platform is primarily attributed to the relatively lagging development of ultrasound therapeutic devices and deficiencies in biomaterial synthesis strategies, drug delivery routes, and biosafety research. Future research should place greater emphasis on the aforementioned unresolved challenges to promote the clinical translation of related technologies and satisfy the clinical requirements of a greater number of patients.

## CRediT authorship contribution statement

**Wei Guo:** Writing – review & editing, Writing – original draft, Project administration, Methodology, Investigation, Conceptualization. **Siying Gao:** Writing – review & editing, Writing – original draft, Methodology, Investigation. **Yiran Hao:** Writing – review & editing, Writing – original draft, Methodology, Investigation. **Zijing Li:** Writing – review & editing, Writing – original draft, Methodology, Investigation. **Haoyuan Hu:** Writing – review & editing, Writing – original draft, Investigation, Conceptualization. **Huijun Wu:** Methodology, Investigation. **Changhao Hu:** Methodology, Investigation. **Xueqin Cheng:** Methodology. **Weiwen Zhao:** Investigation. **Yuxuan Kong:** Investigation. **Hong Jiang:** Funding acquisition, Investigation, Project administration, Resources, Supervision, Writing – review & editing. **Songyun Wang:** Writing – review & editing, Writing – original draft, Project administration, Investigation, Funding acquisition, Conceptualization.

## Declaration of competing interest

The authors declare that they have no known competing financial interests or personal relationships that could have appeared to influence the work reported in this paper.

## Data Availability

No data was used for the research described in the article.
